# Environmental Profile of a Novel High-Amylose Bread Wheat Fresh Pasta with Low Glycemic Index

**DOI:** 10.3390/foods11203199

**Published:** 2022-10-13

**Authors:** Alessio Cimini, Francesco Sestili, Mauro Moresi

**Affiliations:** 1 Department for Innovation in the Biological, Agrofood and Forestry Systems, University of Tuscia, Via S. C. de Lellis, 01100 Viterbo, Italy; 2 Department of Agriculture and Forest Sciences, University of Tuscia, Via S. C. de Lellis, 01100 Viterbo, Italy

**Keywords:** carbon footprint, egg-free fresh pasta, environmental profile, glycemic index, high amylose fresh pasta, mitigation actions, overall weighted damage score, PAS 2050 and ReCiPe 2016 standard methods

## Abstract

To improve glycemic health, a high-amylose bread wheat flour fresh pasta characterized by a low in vitro glycemic index (GI) and improved post-prandial glucose metabolism was previously developed. In this study, well-known life cycle analysis software was used in accordance with the PAS 2050 and mid- and end-point ReCiPe 2016 standard methods to assess, respectively, its carbon footprint and overall environmental profile, as weighted by a hierarchical perspective. Even if both eco-indicators allowed the identification of the same hotspots (i.e., high-amylose bread wheat cultivation and consumer use of fresh pasta), the potential consumer of low-GI foods should be conscious that the novel low-GI fresh pasta had a greater environmental impact than the conventional counterpart made of common wheat flour, their corresponding carbon footprint or overall weighted damage score being 3.88 and 2.51 kg CO_2e_/kg or 184 and 93 mPt/kg, respectively. This was mainly due to the smaller high-amylose bread wheat yield per hectare. Provided that its crop yield was near to that typical for common wheat in Central Italy, the difference between both eco-indicators would be not greater than 9%. This confirmed the paramount impact of the agricultural phase. Finally, use of smart kitchen appliances would help to relieve further the environmental impact of both fresh pasta products.

## 1. Introduction

Owing to its relatively simple production technology, fresh or dried pasta has been regarded a suitable food for enrichment with vitamins and iron by the US Food and Drug Administration since 1944 [1]. Currently, several research groups are attempting to improve the nutritional value of pasta by adding a plethora of bioactive ingredients, such as fiber, polyphenols, minerals, or proteins, of various origin, including several by-products of other production processes, such as brewer’s spent grain, grape or olive pomace, etc. [2]. In this context, numerous breeding programs have tried to increase the amylose level in wheat grain, as there is a correlation between the amount of amylose in kernel and the content of resistant starch (RS) in foods. This starch fraction, being undigested in the small intestine by human a-amylases, but fermented by intestinal microbiota, helps maintain healthy blood sugar levels, and can prevent numerous noncommunicable diseases (i.e., type 2 diabetes, obesity, colorectal cancer, and cardiovascular disorders). Some bread wheat genotypes with an amylose-to-total starch ratio higher than 70% were obtained [3,4,5], and the resulting flours with RS content greater than 10% were used to produce RS-enriched foods, such as dried noodles [6], Japanese noodles, bread and puffed grains [7]. A derived high-amylose line of the bread wheat cultivar Cadenza [3] was grown in open fields in different crop years and, upon milling, yielded a high-amylose bread wheat flour (HABWF), which was used to produce RS-enriched fresh [8,9], and dry [10] pastas. Of these, the fresh pasta made of 100% HABWF was classified as a food with low *in vitro* glycemic index (39 ± 1%) and a resistant starch-to-total starch ratio greater than 14% [9], which allowed the physiological effect of improved glucose metabolism after a meal to be claimed according to EU Regulation 432/2012 [11].

Even if a very large number of papers claim significant improvement in the chemico-physical and nutritional properties of quite numerous fortified pastas, it is still unclear what their real environmental impact is, even when the two basic circular economy concepts detailed by Stahel [12] (that is, product reuse and recycling) were asserted but not proven.

Nowadays, the environmental impact of a food product may be assessed using several different standard methods, as reviewed by Moresi et al. [13]. For instance, the Publicly Available Specification (PAS) 2050 method [14] allows the estimation of the greenhouse gases emitted to produce a given product (i.e., the so-called carbon footprint), while other standard methods account for multiple environmental impact categories, such as acidification, eutrophication, stratospheric ozone depletion, photochemical ozone creation, and so on.

Thus, the main aim of this work was to carry out a business-to-consumer life cycle assessment (LCA) study for the aforementioned high-amylose fresh pasta with attenuated postprandial glycemic response in compliance with the ReCiPe 2016 standard method [15] so as to compare its mid-point environmental profile to that of a default fresh pasta obtained by mixing common wheat flour type 00 with water, identify their main hotspots and suggest a few potentially effective mitigation actions.

## 2. RS-Enriched Fresh Pasta: Definition, Legal Norms, Potential Market, and Environmental Impact

As reported previously [9], the preparation of such a novel fresh pasta was carried out by mixing HABWF with deionized water at a rate of 37 g for every 100 g of flour using a batch mixer for 8 min. The resulting dough was drawn in the form of fresh spaghetti, which was characterized by moisture, total starch and resistant starch contents of 32.6 ± 0.8, 77.0 ± 1.1, and 11.3 ± 0.3 g/100 g wet matter, respectively, an optimal cooking time [16] of 3.0 ± 0.2 min; a post-cooking water uptake of 0.80 ± 0.02 g/g; and a cooking loss of 0.086 ± 0.016 g/g, and in vitro glycemic index (GI) of 39 ± 1% [9].

Such a category of pasta may be commercialized in Italy according to Presidential Decree no. 187/2001 [17], this allowing the production not only of durum-wheat semolina or whole meal dry pastas, but also of egg pastas, special pastas (these including food ingredients other than bread wheat flours), and fresh and stabilized pastas by mixing wheat flour or semolina with water and, in some cases, eggs too, the resulting dough being sheeted, or extruded through bronze or Teflon-coated dies in several different shapes, differently dried and then cooked by boiling or baking.

If fresh pasta is offered for sale in bulk, it must be preserved at temperatures not exceeding +4 °C with a tolerance of 3 °C during transport and 2 °C in the other cases, packed in containers not intended for the final consumer to ensure adequate protection from external agents, and be sold within five days from the date of manufacture [17]. If prepackaged, fresh pasta must have a moisture content (x_W_) not smaller than 24% (*w*/*w*) and a water activity (a_W_) between 0.92 and 0.97, have been submitted to a pasteurization treatment, and be stored at temperatures of 4 ± 2 °C [17]. Thanks to modified atmosphere packaging, the shelf-life of pasteurized fresh pasta may be extended up to 60 days from the date of production. In contrast, stabilized pasta with x_W_ > 20% (*w*/*w*) and a_W_ < 0.92 must be sterilized to enable its transportation and storage at ambient temperatures for as long as 120 days. Such regulations do not apply to pasta made for export from Italy or imported to Italy from other countries, as well as to pastas made in restaurants.

The fresh pasta products examined here were made of common wheat or high-amylose bread wheat flour and water with no addition of table salt or other alkaline compounds, such as sodium and potassium carbonates or sodium hydroxide, and were not fried. For these reasons, they differ from Asian noodles, the most common types of which being white salted noodles, alkaline noodles, instant noodles, buckwheat noodles, rice noodles, etc., even if they were basically made of wheat flour, water and table salt [18].

Both dry and fresh pastas can be classified on the basis of their size as long- (i.e., spaghetti, tagliatelle, ziti, etc.), or short- (i.e., maccheroni, rigatoni, paccheri, fusilli, orecchiette, etc.) style pasta; ingredients used (i.e., egg or egg-free pasta); shapes (i.e., sheets or strips), and stuffing ingredients used in filled pastas (i.e., lasagne, cannelloni, cappelletti, ravioli, tortellini, etc.). Regional variations are also relevant. For instance, in Northern Italy the basic ingredients of fresh pasta are all-purpose bread wheat flour and eggs, while in Southern Italy fresh pasta is made of standard semolina and water mixture.

In 2017, pasta production in Italy approximately amounted to 3.5 × 10^6^ metric tons (Mg), equivalent to a total turnover of around 4.6 billion Euro [19]. It was mainly covered by dry pasta (86% of total), followed by fresh pasta (11%) and frozen pasta (~3%). In 2021, the fresh pasta business in Italy increased to near 892 million Euro with sales up by +5% [20]. Despite the market being dominated by a few national producers, such as Pastificio Rana SpA (San Giovanni Lupatoto, Italy) and Gruppo Fini Spa (Modena, Italy), at the local level consumers still prefer several regional brands, especially in the case of fresh filled pasta [20]. The global market for the latter amounted to 1.6 billion euros in 2020 with an average annual growth rate of 7.2% in the first decade, that slightly decreased to 5.9% in the second one [21]. According to Cognitive Market Research [22], the long-style fresh pasta segment accounted for a major share of 43.82% in 2018 and is expected to reach US$ 487.14 million by 2025 at a compound annual growth rate of about 2%. The growth of the global filled-style pasta segment is expected to be even higher because of the increasing demand from restaurants, hotels, pubs, and households [22]. Under the circumstances, by coupling the present consumer interest, on one side for fresh pasta products and, on the other side, for products bearing low-glycemic index claims, as well as functional and nutraceutical foods, the fresh pasta of concern is expected to receive a positive market response if commercially available.

Owing to the growing interest of the general consumer towards the environmental impact of the foods and beverages daily consumed, the major Italian pasta manufacturers (i.e., Barilla Group and De Cecco SpA) have started to assess the environmental impact of dry pasta production using the *Environmental Product Declaration* methodology [23], as reported online at https://www.environdec.com/ (12 October 2022). The cradle-to-grave environmental impact of dry pasta production was originally assessed by Bevilacqua et al. [24]. After that several life-cycle assessment (LCA) studies were carried out, as reviewed by Renzulli et al. [25]. Some of these were of the business-to-business type [26,27,28,29], while others were cradle-to-grave types [24,30,31,32,33,34,35]. According to Barilla [36], the business-to-consumer carbon footprint (CF_B2C_) of 1 kg of durum wheat semolina dry pasta in paperboard boxes was of the order of 1.9 or 3.0 kg CO_2e_, depending on the use of a gas or electric stove, respectively. Durum wheat cultivation represented 32 or 20% of CF_B2C_, while pasta cooking, embodying 31 or 56% of CF_B2C_, was the most impacting phase of the overall life cycle of dried pasta. Moreover, the cradle-to-grave environmental profile of an organic pasta production chain, estimated in compliance with the *Product Environmental Footprint* standard method [37] and specific category rules for dry pasta [38], was characterized by an overall weighted eco-indicator of 195 μPt/kg, this being about 40% higher than that of conventional pasta (~141 μPt/kg) [14]. Both eco-indicators were mainly affected by the field phase (57% vs. 45%, respectively) and then by the pasta cooking one (23% vs. 30%).

A specific search using the Scopus database retrieved 422 documents when using the research terms *environmental profile* AND *life cycle assessment* AND *fresh pasta* in the title, abstract, and keywords research fields. Except for a couple of papers [39,40] dealing with the energy and steam consumption in an artisanal fresh pasta industry with an overall capacity of 68 Mg/year, no document has so far dealt with the environmental profile of any fresh pasta type. This was also confirmed by using the search engine Google or Research Gate. Thus, this LCA study will be the first one dealing with the carbon footprint and environmental profile of a novel low-GI fresh pasta as compared to those of a conventional fresh pasta.

## 3. Methodology

This work was based on the Life Cycle Assessment procedure defined by ISO norms no. 14,040 [41] and 14,044 [42], which included the following stages: Goal and scope definition, inventory analysis, impact assessment, and interpretation of results.

### 3.1. Goal and Scope Definition

The goal for this study was to develop a Life Cycle Assessment model to assess the environmental profile of a low-GI fresh pasta produced from a medium-sized fresh pasta factory, as well as to identify its life-cycle hotspots.

**Figure 1:**
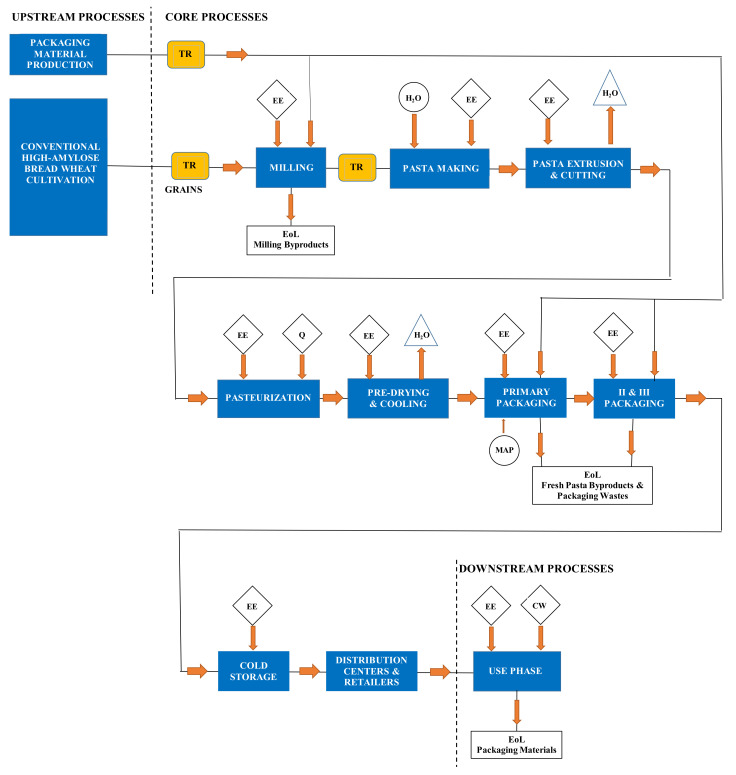
Pasteurized high-amylose fresh pasta system boundary, where the main processes are subdivided into upstream, core and downstream ones: CW, cooking water; EE, electric energy; EoL, end of life; H_2_O, process water; MAP, modified atmosphere packaging; Q, thermal energy; TR, transport.

The functional unit was assumed as 1 kg of high-amylose bread wheat (HABW) fresh pasta, as packed in 0.5-kg modified atmosphere polyethylene (PE) bags, while the system boundary comprised the upstream (from cradle to gate), core (from gate to distribution centers, DCE), and downstream (from DCE to grave) processes as shown in Figure 1.

The upstream processes consisted of HABW cultivation, production of seeds, fertilizers, pesticides, auxiliary products (i.e., lubricants, detergents for cleaning, etc.), and packaging materials, and accounted for the electricity and fuel used in the agricultural treatments. The core processes included the bulk transportation of HABW grains to the mill to obtain a HABW flour. The latter, as well as all packaging materials, were transported to the fresh pasta factory, where fresh pasta was manufactured, pasteurized, packed in modified atmospheric conditions, and stored in refrigerated cells. Palletized fresh pasta was then transported to the distribution centers and retailers by means of refrigerated lorries. All processing wastes and by-products underwent municipal solid waste (MSW) recovery and disposal. Finally, the downstream processes accounted for the consumer use of fresh pasta, and recovery and disposal of all post-consumption wastes as MSW.

The production of capital goods (machinery, etc.), as well as their cleaning and disposal, any personnel travel, and the transport of consumers to and from points of purchase were excluded from the system boundary, as suggested by Sections 6.5 and 6.4.4 of PAS 2050 standard method [14]. Moreover, the LCA was referred to the year 2021, while the process technology underlying the datasets used in this study reflected the process configurations typical of fresh pasta processing on an industrial scale in the reference year. The primary data for high-amylose wheat flour production and milling byproducts were provided by Grandi Molini Italiani SpA (Venice, Italy) according to some grinding tests on laboratory- and pilot-scale mills. The primary data for fresh pasta production and distribution, as well as the formation and disposal of by-products and packaging wastes, were provided by the company Nuova Tortuovo Srl (Rome, Italy: https://www.tortuovo.info/) (12 October 2022), that has been operating in the fresh pasta sector since 1940 and is currently producing around 8,100 Mg of fresh pasta per year. The secondary data were extracted from the Ecoinvent v. 3.8 database using the allocation, cut-off, EN15804 system model [43], which was incorporated into the LCA software Simapro 9.3.0.3 (Prè Consultants, Amersfoort, The Netherlands), and other technical reports, as detailed below.

Both primary and secondary data were used to identify six different product stages (i.e., processes, assembly, reuse waste scenarios, end-of-life scenarios, and life cycles) and thus create the fresh pasta network using the software SimaPro 9.3.0.3. Moreover, the parallel life cycles of the primary, secondary, and tertiary packaging materials, and tertiary package (i.e., pallet) were included to incorporate their end-of-life scenarios. These product stages are summarized in the electronic supplement (Table S1) and will be detailed below.

### 3.2. Life Cycle Inventory Analysis

#### 3.2.1. High-Amylose Bread Wheat Production

A derived high-amylose line of the bread wheat cultivar Cadenza [3] was grown in open fields at two agricultural farms according to the crop management conditions described in Table 1. In both farms standard cultivation methods were used in conjunction with reduced tillage at the experimental farm of the University of Tuscia (Farm 1) or direct seed drilling in the stubble of the previous crop at Farm 2. Nitrogen fertilization was split into three applications: the first one was given before sowing as diammonium phosphate (200 kg/ha), the second one when the first node was detectable above ground as urea (150 kg/ha), and the third one 25 days later as ammonium nitrate (150 kg/ha).

Table S2 in the electronic version of supplementary material shows the input and output data regarding the conventional production of high-amylose bread wheat at the Massimo Filzi farm (Farm 2), as referred to a nominal area of 1 ha, together with the GHG emissions from fertilized soils as estimated according to the recently updated IPCC Guidelines [44]. As indicated by the category rules EPD^®^ [45], an allocation procedure of the economic type was applied, this involved an allocation factor of 92.5% for HABW grains and 7.5% for wheat straw. Table S3 in the electronic supplement describes the inventory associated with the HABW cultivation phase.

#### 3.2.2. High-Amylose Bread Wheat Flour Production

**Figure 2:**
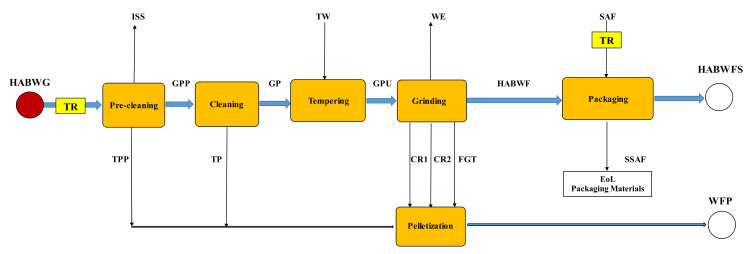
Block diagram of the high-amylose bread wheat milling process carried out in the pilot-scale miller at Grandi Molini Italiani SpA (Venice, Italy). All symbols are listed in the Nomenclature section.

During milling, HABW grains were pre-cleaned, cleaned, tempered, and then ground to break down the endosperm into fine particles (white flour) and several byproducts, such as bran, middlings, meal, germ, and groats. Figure 2 shows the block diagram of the HABW milling process, where the symbols used for all the streams accounted for are listed in the Nomenclature section.

Table 2 shows the main results of the grinding tests performed in laboratory- and pilot-scale mills at Grandi Molini Italiani SpA (Venice, Italy). The HABW flour yield was around 56% in the laboratory-scale mill, but slightly reduced to 54% in the pilot-scale one. An optimal moisture content of 15.5% (*w*/*w*) of tempered clean grains was also assessed in a few pilot-scale tests.

Table S4 in the supplementary material shows the reconstructed material balance of the grinding tests performed in the pilot-scale mill together with all pre-cleaning, cleaning, wet grain, flour, and by-product yields. By setting the moisture contents of HABW grain to 11% (*w*/*w*) (cf. Table 1) and GPU to 15.5% (*w*/*w*) and keeping the dry matter content of GPU as equal to that of the output streams of the grinding step (CR1, CR2, FGT, and HABWF), it was possible to estimate their moisture content as equal to 12.88% (*w*/*w*). Thus, the HABWF yield was about 54% of the clean wet grains (i.e., 53.4% of the input raw grain), while that of wheat feed pellets (WFP) with a moisture level of 12.6% (*w*/*w*) was nearly 48.5% of raw HABW grains. Such estimates were used not only to reconstruct quite accurately the experimental data collected in the laboratory-scale mill (Table 2), but also to calculate the material balance of the milling step of the HABW grains harvested from a nominal fertilized soil area of 1 ha (i.e., 1500 kg), as shown in Table S5. The high-amylose bread wheat flour was finally packed in 25-kg kraft paper bags, each one weighing about 115 g, and then dispatched to the fresh pasta industry, the inventory associated with this production being shown in Table S6. The wheat feed pellets were used as cattle feed.

The impact of grain milling was allocated according to an economic-based criterion using an allocation factor of 84% for wheat flour and 16% for by-products, as suggested by the category rules of the Product Environmental Footprint [38].

Finally, the specific consumption of lubricating oil and detergents was assumed as coincident with that previously registered in an industrial mill [30], that is 0.0021 L of lubricating oil and 0.0017 L of detergent per Mg of grains milled.

Table S7 describes the inventory associated with HABW milling phase.

When milling conventional bread wheat, the extraction rates were of 72–75% for flour and 25–28% for wheat feed pellets [46].

#### 3.2.3. Fresh Pasta Making, Pasteurization, Pre-drying, and Cooling

**Figure 3:**
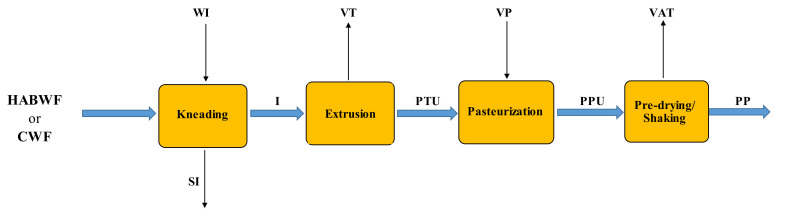
Flowchart of the fresh pasta making, pasteurization, and pre-drying processes using HABW flour or type 00 common wheat flour (CWF). All symbols were listed in the Nomenclature section.

Figure 3 shows the block diagram of the pasta making process. The dough (I) was prepared by mixing the raw material, that is high-amylose bread wheat flour (HABWF) or common wheat flour type 00 (CWF), with water directly in the mixer. The hydration ratio was equal to 0.37 kg of water per kg of HABWF [32], or to 0.25 kg of water per kg of CWF, as generally used in the fresh pasta industry mentioned above. The dough waste (SI) was assumed as equal to 0.4% (*w*/*w*) of the dough produced (I), as measured in industrial pasta making tests [30]. The dough (I) was then transferred to the extruder and forced through dies to obtain pasta strands of different shapes and sizes (PTU). Despite water cooling jackets allowing the heat produced during the extrusion process to be removed to maintain the dough temperature near to 50 °C, wet pasta (PTU) reduced its moisture content to 31% (*w*/*w*). Extruded wet pasta strands were conveyed to the pasteurizer by means of stainless-steel mesh conveyor belts. Injection of saturated steam allowed such strands to be heated up to 87 °C for about 3 min to reduce the bacterial population. Pasteurized fresh pasta was then loaded into a pre-dryer to reduce its humidity to 24% (*w*/*w*) in compliance with the provisions of art. 9 of Presidential Decree no. 187/2001 [17]. Thereafter, a continuous ventilation process enabled pasteurized fresh pasta to be cooled to 4–6 °C. Table S8 shows the partial and total material balances of the fresh pasta making, pasteurization, and pre-drying steps.

Table S9 shows the inventory associated with the fresh pasta making phase.

#### 3.2.4. Fresh Pasta Packaging 

Five hundred grams of pasteurized fresh pasta at 4–6 °C were packed in a food-grade polythene (PE) bag. The packaging machine firstly eliminated all the air present, then filled the bag with a mixture of food-grade N_2_ and CO_2_, both in the liquid state, in a volumetric ratio of 3/1, and finally sealed it. This modified atmosphere packaging allowed the storage time of fresh pasta in the fridge to be extended up to 60 days. The overall specific consumption of such gases was rounded up to 10 g of N_2_ and 5 g of CO_2_ per kg of fresh pasta packed. The secondary package comprised a few 0.5-kg PE bags assembled in a carton made of recycled cardboard, the latter being then labeled and sealed with a scotch tape strip. The tertiary one consisted of a 120×80-EPAL wood pallet over which different layers of cartons were stacked, tightened with 30-μm stretch-and-shrink PE film, and labeled with two adhesive paper tags.

**Figure 4:**
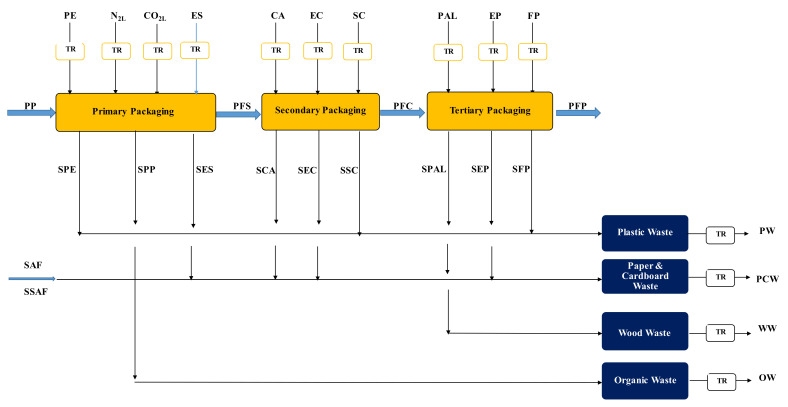
Schematic diagram of the packaging process used for the pasteurized fresh pasta under study. All symbols were listed in the Nomenclature section.

Figure 4 shows the block diagram for the packaging process examined in this work, showing also all the solid wastes generated, while Table 3 gives all the details about the primary, secondary, and tertiary packages used.

By accounting for the average percentage of fresh pasta and packaging materials discarded during the operation of the reference fresh pasta industry in the year 2021 (cf. Table S10), it was possible to calculate the total material balance of the packaging process for the pasteurized low-GI fresh pasta under study, as shown in Table S11.

Moreover, the specific consumption of lubricating oil and detergents for fresh pasta making and packaging was assumed as coincident with that previously registered in an industrial dry pasta plant [30], that is 0.0295 L of lubricating oil and 0.0156 L of detergent per Mg of fresh pasta produced.

Pelletized fresh pasta was kept in cold cells at temperatures not exceeding +5 °C for 3 to 10 days before being transported to a few distribution centers.

The impact of fresh pasta making was fully allocated to fresh pasta, the discarded fraction of which (cf. Table S10) being disposed of as organic waste.

The inventory associated with all packaging items is shown in Tables S12–S17.

The assemblies of primary, secondary, and tertiary packages are detailed in Tables S18–S20, while those of primary and secondary packaging and primary, secondary, and tertiary packaging in Tables S21 and S22. Finally, the assembly of dry pasta is described in Table S23.

#### 3.2.5. Logistics of Input and Output Materials

Table 4 shows the logistics of the input/output materials with the type and load of the means of transport used and overall distance travelled from the places of production to those of use/delivery, as mainly derived from the fresh pasta processing plant of reference. Palletized fresh pasta was loaded on isothermal lorries to keep the cold chain unaltered till the distribution centers and points of sale, where pasteurized fresh pasta bags were displayed in refrigerated counters at about +5 °C for 7–10 days. Such data were included in the product stages of the fresh pasta network mentioned above.

#### 3.2.6. Energy Sources

The energy resources used for producing fresh pasta were electricity and natural gas. Electricity was used to operate wheat mills, mixers, extruders, conveyor belts, fans, packaging machines, refrigerators, lighting, heating, and air conditioning of the production plant premises, etc. Steam produced by a methane gas boiler was used for pasteurizing fresh pasta. The electricity was absorbed by the Italian medium-voltage grid, while the methane gas by the national distribution network.

The grinding tests (Table 3), carried out in a pilot-scale mill using a 7.5-Mg sample of high-amylose bread wheat grains at Grandi Molini Italiani SpA (Venice, Italy; www.grandimolini.it; 12 October 2022), involved an electricity consumption of about 1100 kWh, equivalent to 147 Wh/kg of grain milled, such specific consumption yield falling within the range of values detected by Carlsson-Kanyama and Faist [47]. Such energy consumption was included in the HABW flour production step (cf. Table S7).

Concerning the production, packaging and refrigerated storage of pasteurized fresh pasta, the electricity and natural gas consumption data provided by Nuova Tortuovo Spa (Rome) were referred to the annual production of 8100 Mg of fresh pasta and resulted in an overall electricity and natural gas requirement of about 110 and 6 kWh per Mg of fresh pasta produced, respectively. Such estimates were by far lower than those reported for an artisanal fresh pasta factory in Molise with a production capacity close to 68 Mg/year [39,40], these yielding a specific consumption of electricity and natural gas of 186 and 1034–1340 kWh/Mg of fresh pasta produced, respectively. Such thermal energy needs appeared to be definitively overestimated since they were even higher than those (~441 kWh/Mg) registered in an industrial plant with an annual capacity of approximately 130,000 Mg of durum wheat semolina dry pasta [30]. In this work, the electric and thermal energy needs mentioned above were cautiously assumed as equal to 200 and 20 kWh/Mg, respectively, with the final aim of assessing the sensitivity of the environmental impact categories selected to their relative variation. Such energy consumption needs were included in the HABW fresh pasta production step (cf. Table S9).

#### 3.2.7. Fugitive Emissions of Refrigerant Gases

The overall nominal power of the refrigerated cells installed at the reference fresh pasta plant (with a total capacity of 8100 Mg of fresh pasta per year) was approximately 30 kW. The refrigeration circuit was loaded with ~105 kg of a non-toxic and non-flammable ternary refrigerant mixture (R404a) consisting of (44 ± 2)% pentafluoroethane (R-125), (52 ± 1)% of 1, 1, 1-trifluoroethane (R143a) and from (4 ± 2)% 1,1,1,2-tetrafluoroethane (R134a) [48]. Despite R404a being widely used in commercial refrigerators/freezers with a Global Warming Potential of 3922 kg CO_2e_/kg and a zero Stratospheric Ozone Depletion Potential, it can no longer be used in new equipment, but it can be recharged in existing equipment until 2030 [48]. By assuming a refrigerant leakage of about 5% per year [49], the overall fugitive emissions would amount to ca. 0.65 mg per kg of fresh pasta packaged and were included in the fresh pasta production step (Table S9).

#### 3.2.8. Consumer Use

Pasteurized fresh pasta is preserved in domestic refrigerators by end users. The energy efficiency of the home refrigerators purchased in Italy is increasing, even if the percentage fraction of energy class A^+++^ refrigerators is still 15.4%, a fraction higher than that recorded in France (10.7%), but significantly lower than that registered in Germany (36.7%) and Spain (29.8%) [50]. Based on estimates by Enea [51], the weighted annual energy consumption of home refrigerators of energy classes A^+++^, A^++^, A^+^, A, and B acquired in 2018 would be near to 200 kWh/year. In this work, the percentage energy class distribution of the home fridges in use in Italy being unknown, the following was assumed according to the most recent category rules for pasta [52]:

- The energy consumed by a class A refrigerator was ~300 kWh/year.
- The number of foods and beverages averagely stored in a home refrigerator over a year-time basis was near to 10 kg. Thus, the daily specific energy consumption was equal to [300 kWh/(365 days × 10 kg) =] 0.082 kWh/(day kg).
- The average residence time of fresh pasta in the refrigerator at home was half of its claimed shelf-life, that is 30 days in this specific case.

In the circumstances, the average electric energy consumption resulting from fresh pasta preservation in home refrigerators would be equal to 2.46 kWh per kg of fresh pasta.

To cook 1 kg of fresh pasta, 10 L of boiling water laced with 100 g of table salt are usually used [52]. Since the default energy requirement is around 0.18 kWh for boiling 1 kg of water, and 0.05 kWh/min for cooking 1 kg of pasta and the optimal cooking time of high-amylose bread wheat fresh pasta was about 3.5 min, as previously determined according to the ISO 7304-1 method [16] by Cimini et al. [9], the overall cooking energy need amounted to 1.975 kWh, of which (0.18 kWh/kg × 10 kg =) 1.80 kWh to boil 10 L of water and (0.05 × 3.5 =) 0.175 kWh to cook 1 kg of the fresh pasta under study. Moreover, in the European Union 83% of domestic kitchens use gas cookers, while the remaining 17% electric ones [38] by withdrawing natural gas or electricity from the national network or low-voltage grid.

The inventory of the fresh pasta consumption step is shown in Table S24.

#### 3.2.9. Disposal of Processing and Post-Consumer Wastes

All wastes formed during the life cycle of fresh pasta were collected in containers of different colors according to the municipal solid waste (MSW) collection process, as reported below:

- Dough (SI), pasteurized pasta (SPP), and cooked pasta wastes were collected in the bins for the organic fraction of municipal solid waste (MSW).
- Packaging materials (i.e., paper bags, PE bags, cartons, adhesive paper labels, scotch tape, and PE thermo-retractile film) discarded during the primary packaging of wheat flour or fresh pasta, as well as the secondary and tertiary packaging of fresh pasta during its manufacture or storage at distribution centers, retailers, and consumers, were fractionated and amassed in the bins for paper and cardboard or plastic waste.
- The wooden pallets, damaged during tertiary packaging or management at the distribution centers, were collected and returned to the Euro pallet managing center to be repaired and made newly available to the fresh pasta factory gate, while unrepairable aliquots were gathered in the bins for wood waste.

The percentage fraction for any packaging item discarded during the life cycle of fresh pasta is shown in Table S10 in the electronic supplement.

Organic and packaging waste were disposed of according to the Italian scenarios for the overall management of municipal solid waste (MSW) in 2019 [53], as reported in Table 5. In 2019, 31% of the organic fraction was landfill, 18% incinerated and 51% recycled [54,55]. As suggested by EPD^®^ [52], 25.5% of the recycled organic fraction was composted and the remaining 25.5% anaerobically digested. Finally, unsorted municipal solid waste, such as ISS, was landfilled (52.6%) and incinerated (47.4%), as estimated by Legambiente [56]. Such disposal scenarios were described in Tables S25–S27.

Upon cooking, each gram of the high-amylose bread wheat fresh pasta absorbed 0.70 ± 0.04 g of water [9]. Default food loss rate at consumer was assumed to be 2% of cooked fresh pasta [38,52]. However, Last Minute Market, a spin-off of the University of Bologna (Italy), in cooperation with Barilla Group SpA (Parma, Italy) observed that cooked pasta waste at the domestic and hospitality sector was by far greater ranging from 10 to 40%, while in school catering its average rate was near to 25%, probably because the excessive portions prepared were mostly unconsumed [57]. The effect of this scenario will be considered in the parametric sensitivity analysis.

Concerning the disposal of industrial wastewaters, their specific formation yield was assumed as equal to 72 L per Mg of fresh pasta, as derived from previous estimates in an industrial dry pasta factory [30]. At the consumer level, the residual pasta water was generally drained into kitchen sinks and thus disposed of in the municipal sewer system. Whereas such disposal was found to exert a negligible contribution to the overall carbon footprint of pasta cooking [58], in this work it was accounted for assessing its effect on other environmental impact categories, such as eutrophication.

To account for the end of life of the primary, secondary and tertiary packaging material wastes associated with 1 kg of packed fresh pasta, the waste scenarios were allocated on a mass-based criterion, as reported in Tables S28–S30. In particular, the end of life of the wooden pallet included 99.8% of pallet reuse and 0.2% of pallet disposal (Tables S30 and S32). In these stages the final transport of packaging wastes to the waste collection center (WCC) was included.

#### 3.2.10. Life Cycle of Fresh Pasta

The life cycle of fresh pasta linked the assembly of fresh pasta to its distribution logistics with refrigerated lorries, consumer use, as well as the life cycle of the primary, secondary and tertiary packaging materials, as shown in Table S33. The latter is described in Table S34 and was linked to the life cycle of the wooden pallet, as detailed in Table S35. Both these life cycles allowed their relative assembly stages to be related to their corresponding end-of-life disposal scenarios (Tables S30 and S31).

### 3.3. Impact Assessment

The impact assessment was carried out using the Publicly Available Specification (PAS) 2050 [14] and ReCiPe 2016 [15] standard methods, which were embedded in the software SimaPro 9.3.0.3 (PRé Consultants, Amersfoort, The Netherlands). Whereas the former refers to a single environmental area of protection only, the latter accounts for more than one impact categories at midpoint and endpoint levels. Each generic impact category (IC_j_) was estimated by summing up the release to air, water or soil (*Ψ_i,j_* expressed in mass, energy, mass-km basis) associated to the system boundary times its corresponding characterization factor (*F_i,j_)* as:

(1) $${\mathrm{IC}}_{j}=\sum_{i}(\Psi_{i,j}F_{i,j})$$

In particular, the characterization factors of the single environmental impact category (i.e., global warming) considered by the PAS 2050 standard method coincided with the 100-year time-horizon global warming potentials, as extracted from IPCC [59]. On the contrary, the updated ReCiPe 2016 method [15] included the following 18 midpoint impact categories, their reference substance being shown in brackets: *global warming* (kg CO_2e_); *stratospheric ozone depletion* (kg CFC-11_e_); *ionizing radiation* (kBq ^60^Co_e_); *fine particulate matter formation* (kg PM_2.5e_); *ozone formation-human health* and *-terrestrial ecosystems* (kg NO_xe_); *terrestrial acidification* (kg SO_2e_); *freshwater* (kg P_e_) and *marine* (kg N_e_) *eutrophication*; *terrestrial*, *freshwater*, and *marine eco-toxicity* (kg 1,4-DCB); *human carcinogenic* and *non-carcinogenic toxicity* (kg 1,4-DCB); *land use* (m^2^ yr crop_e_); *mineral* (kg Cu_e_) and *fossil* (kg oil_e_) *resource scarcity*; and *water consumption* (m^3^). Such mid-point impact categories can be aggregated to three endpoint indicators: (i) damage to human health (HH), expressed in DALY; (ii) damage to ecosystems (EQ), expressed in Loss of species during a year; (iii) damage to resource availability (RA), expressed in US$2013 to quantify the extra costs involved for future mineral and fossil resource extraction. These damage categories were then normalized with respect to the global population and finally might be grouped into the individualistic, hierarchic, or egalitarian perspective, according to the “Cultural Theory” [60]. In this work, the hierarchic perspective was selected to estimate an overall weighted damage score (OWDS), since such a perspective is regarded as the most balanced one between future and present impacts, and risks and benefits [15].

### 3.4. Sensitivity Analysis

The uncertainty in the input and output data of the above LCA model was specifically assessed by resorting to the Monte Carlo analysis [61], embedded in the LCA software SimaPro used here, once assigned a fixed number (i.e., 1000) of iterations. The sensitivity of both the environmental eco-indicators was also assessed as follows:

(i) The emission factors characterizing each input data, as well as the recycling of any post-consumer waste, were extracted from the same EcoInvent v. 3.8 database when using the system model Allocation at the point of substitution, simply known as APOS system model [62]. The APOS system model follows the attributional approach in which burdens are attributed proportionally to specific processes. In this way, recyclable materials are linked to the input side of the activities producing them with a negative sign, this being equal to a CO_2e_ credit.
(ii) The specific electric and thermal energy consumption yields during fresh pasta production were increased by +100% with respect to the default conditions (i.e., 200 kWh/Mg and 20 kWh/Mg, respectively).
(iii) The default cooked pasta waste of 2% of cooked pasta [47] was enhanced by a factor of 10, as detected by Barilla [57].

In this way, it would be possible to assess the relative variation of the generic B2C eco-indicator (i.e., ΔCF_B2C_ or ΔOWDS_B2C_) with respect to the corresponding reference value (CF_B2C,R_ or OWDS_B2C,R_) as divided by the relative variation (ΔX_i_) of the independent parameter X_i_ accounted for, with respect to the corresponding reference value (X_iR_), this ratio (m_i_) elucidating how each eco-indicator is sensitive towards the relative variation of X_i_ accounted for.

## 4. Results and Discussion

### 4.1. The Carbon Footprint of High-Amylose Bread Wheat Grain and Related Fresh Pasta

The carbon footprint of high-amylose bread wheat was estimated based on the fertilizer, pesticide, seed density, and diesel fuel inputs and resulting yield factors for above and below ground biomasses, these being summarized in Table 1, and accounted for the on-field emissions from fertilized soil (Table S2).

**Figure 5:**
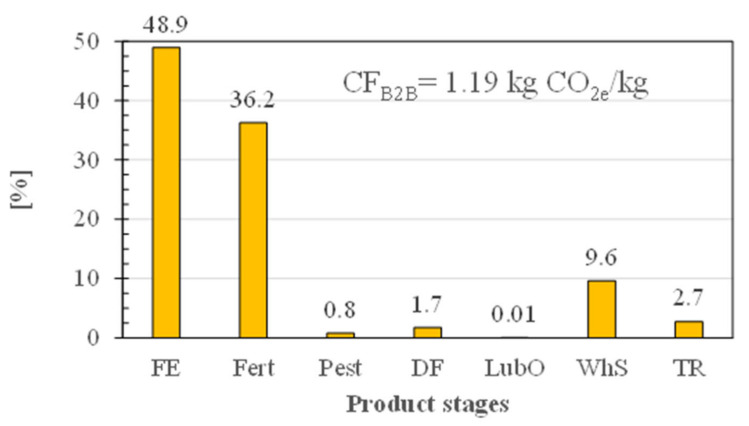
Percentage contribution of the different life cycle product stages to the business-to-business carbon footprint (CF_B2B_) of high amylose bread wheat, as well as its overall score: FE, field emissions; Fert, fertilizers; Pest, pesticides; DF, diesel fuel; LubO, lubricating oil; WhS, wheat seeds; TR, transportation.

As shown in Figure 5, the carbon footprint of HABW grains at farm gate (CF_B2B_) amounted to 1.19 kg CO_2e_/kg. The on-field direct and indirect N_2_O and CO_2_ emissions represented the primary spot (48.9%), while the production of synthetic fertilizers (i.e., ammonium nitrate, diammonium phosphate and urea) the secondary spot (36.2%), and grain seed cultivation the third one (9.6%), the transportation stage the fourth one (2.7%), and diesel fuel consumption for management practices the fifth one (1.7%), the latter being a consequence of the reduced-tillage management of soil applied (Table 1).

Once the default triangular and/or normal distribution uncertainty range for the main materials and processes had been accounted for, the well-known Monte Carlo analysis, embedded in the software SimaPro 9.3.0.3, was used by running one thousand trials to determine all the possible CF_B2B_ outcomes and the probability that they will occur. The CF_B2B_ scores appeared to be normally distributed with a mean value of 1.17 ± 0.26 CO_2e_/kg and a coefficient of variation of 22%. Thus, it was definitively higher than that (0.69 kg CO_2e_/kg) associated with the conventional wheat grain production (Wheat grain {RoW}Cut-off, S), as extracted from the EcoInvent v. 3.8 database, this process averaging local annual production volumes in Germany, Spain, France and USA and including all inputs (i.e., seeds, mineral fertilizers, pesticides and irrigation water), machine operations (i.e., soil cultivation, sowing, fertilization, irrigation, weed control, pest and pathogen control, combine-harvest and transport from field to farm), direct field emissions, and drying of grains at the farm gate.

Owing to a dry season in 2020 and to a three-day frost (at −3 °C) in April 2021, the average crop yields per hectare obtained in the experimental farms (Table 1) were quite lower than the average bread wheat yields (7.26 Mg/ha) generally achieved in Central Italy [63], this making the CF_B2B_ of HABW as great as that shown in Figure 5. In fact, by setting the average crop yield for HABW as equal to the average one mentioned above, the CF_B2B_ of HABW would reduce to ~0.28 kg CO_2e_/kg.

Table 6 shows that the business-to-business CF_B2C_ of 1 kg of HABW fresh pasta amounted to about 3.91 kg CO_2e_/kg, this accounting for the contribution of all the life cycle phases, which were ranked in the following manner: field cultivation (1624 g CO_2e_/kg), use phase (1554 g CO_2e_/kg), packaging material manufacture (335 g CO_2e_/kg), transportation (122 g CO_2e_/kg), pasta production and modified atmosphere packaging (95 g CO_2e_/kg), HABW milling (82 g CO_2e_/kg), end of life of packaging materials (68 g CO_2e_/kg), and disposal of cooked fresh pasta waste (19 g CO_2e_/kg).

The carbon footprint of the low-GI fresh pasta was then compared to that of an analogue fresh pasta made of type 00 common wheat flour (CWF) only, which was produced via the same pasta making process already described under the following conditions:

(a) Common wheat grains were cultivated in Central Italy with an average crop yield of 7.26 Mg/ha, the inventory of such a phase being described in the electronic supplement (Table S36).
(b) Type 00 common wheat flour was produced and packed in 25-kg Kraft paper bags in an industrial mill located in Spoleto (Italy), as indicated by the reference fresh pasta industry, the average flour yield being about 73% of raw common wheat grains. Table S37 describes the inventory associated with the milling phase.
(c) About 0.25 kg of water per kg of common wheat flour was used to prepare the dough, as indicated by the reference fresh pasta industry. Table S38 shows the inventory associated with the fresh pasta production step using type 00 common wheat flour. Moreover, the water uptake by cooked pasta was assumed as equal to 0.74 ± 0.02 g H_2_O per g raw fresh pasta, as determined by Cimini et al. [9].

As shown in Table 6, the CF_B2C_ of such conventional fresh pasta amounted to about 2.51 kg CO_2e_/kg with a far lower impact of the cultivation and milling phases as a direct consequence of the greater crop (7.26 vs. 1.5 Mg/ha) and flour (0.73 vs. 0.54 kg/kg raw grains) yields. In these conditions, the primary hotspot of conventional fresh pasta was represented by the use phase, this covering as much as 62% of the overall B2C carbon footprint. Even if the fresh pasta making process lacks the final drying step to reduce the moisture content of dried pasta to as low as 12.5% (*w*/*w*) according to DPR [17], fresh pasta preservation in industrial, retailing, and domestic refrigerators, as well as refrigerated transportation, make its B2C carbon footprint higher than that of conventional (1.88 kg CO_2e_/kg) and organic (2.05 kg CO_2e_/kg) durum wheat semolina dry pasta [31].

### 4.2. The Environmental Profile of Low-GI Fresh Pasta

Table 7 shows the midpoint characterization factors of one functional unit of HABWF fresh pasta as resulting from the framework provided by the ReCiPe 2016 standard method.

As shown by the data in bold types, the agricultural phase exerted a major effect on the midpoint impact categories of *land use* (97.9% of its overall impact), *stratospheric ozone depletion* (96.4%), *marine eutrophication* (90.9%), *ozone formation—terrestrial ecosystems* (81.8%), *terrestrial acidification* (72.3%), *freshwater eutrophication* (71.2%), *mineral resource scarcity* (70.3%), *ozone formation—human health* (69.2%), *water consumption* (67.3%), *fine particulate matter formation* (67.1%), *global warming* (42.3%), and *terrestrial ecotoxicity* (39.5%). By contrast, the consumer use phase primarily affected the midpoint impact categories of *freshwater ecotoxicity* (61.8%), *marine ecotoxicity* (60.0%), *ionizing radiation* (57.9%), *fossil resource scarcity* (~46.6%), *human carcinogenic* (44.7%) and *non-carcinogenic* (41.4%) *toxicity*. The packaging material production was generally the tertiary hotspot for the great majority of the midpoint impact categories, but the secondary one for the *ionizing radiation* one (19.8% of total).

**Figure 6:**
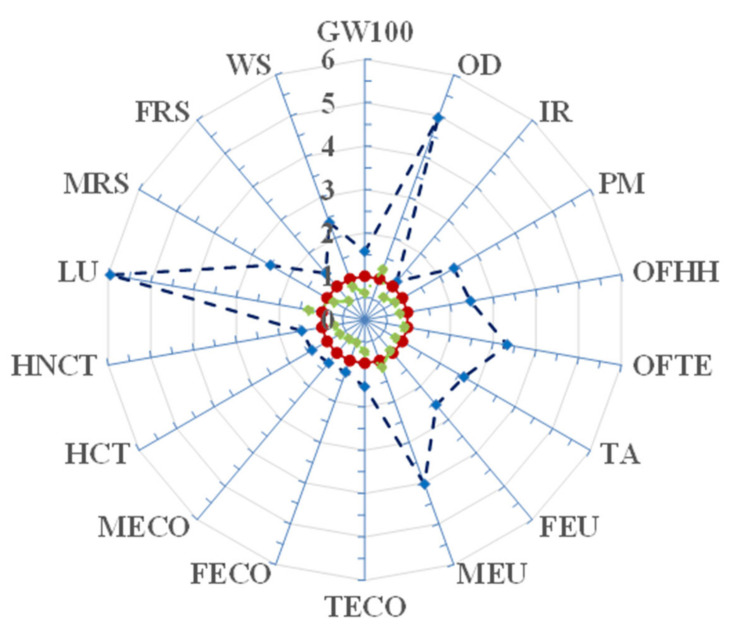
Radar diagram of the B2C environmental profiles of high-amylose fresh pasta either in the default conditions (dark blue broken line) or in the three mitigation options considered below (green dash-dotted line) as referred to that of the conventional common wheat flour counterpart (red dotted line). For the acronyms of the 18 impact categories refer to Table 7.

Figure 6 compares the environmental profile of such low-GI fresh pasta to that of a conventional fresh pasta. It can be noted that the ratio between the scores of each environmental impact category ranged from as low as 1.2 in the case of ionizing radiation to as high as 6.0 in the case of land use, being near to 1.6 for global warming. Actually, the scores of the latter (3.99 and 2.58 kg CO_2e_/kg) were slightly higher than those shown in Table 6 for the 100-yr time horizon global warming potentials used by the ReCiPe 2016 method that were extracted from the 2013 version of the IPCC method [64] instead of the 2021 version [59], which was used in Table 6.

The ReCiPe 2016 standard method grouped all the 18 midpoint impact categories into three damage categories (DC), to underline the environmental compartments damaged by fresh pasta in its life cycle (Table 8). The damage impact on human health (HH) and ecosystem quality (EQ) mainly derived from the cultivation phase, as shown by the data in bold types in Table 8. The use phase mostly damaged the compartment of resource availability (RA).

To complete the assessment to the end-point approach, the single score (SS_z_) of each damage category (DC_z_) was firstly normalized and then aggregated (Table 8). The estimated single score (OWDS) amounted to 184 ± 20 mPt, about 83.9% of which represents the damage on human health, 14.7% on ecosystem quality, and the remaining 1.4% on resource availability. The field phase covered 59.7% of OWDS, while the use phase and packaging material manufacture represented 26.5% and 7.4% of OWDS, respectively. By contrast, the overall weighted damage score for conventional fresh pasta was about a half of the former, that is 93.0 ± 1.5 mPt/kg, 91% of which being covered by the damage on HH, 7% to that on EQ, and remaining 2% to that on RA.

### 4.3. Sensitivity Analysis

To account for all credits potentially deriving from the recycling of renewable and non-renewable materials, the above LCA model was newly run by referring to the characterization factors extracted from the EcoInvent v. 8 database when using the APOS system model, and to the waste disposal rates shown in Table 5. Table 6 lists the GHG emissions associated to each life cycle phase. The CO_2e_ credits deriving from the high recycling rates of paper and cardboard waste (i.e., ~81%), as well as plastic packaging ones (~46%), had the effect, on one side, of reducing the GHG emissions associated with packaging material production by 1.3% (that is, from 335 to 291 g of CO_2e_ per kg of fresh pasta), and, on the other side, that of halving the GHG emissions associated with their end-of-life disposal (that is, from 68 to 33 g CO_2e_/kg). Altogether, the B2C carbon footprint reduced by about 2% from 3.91 to 3.83 kg CO_2e_/kg. However, when the uncertainty distributions for these carbon footprint scores, as derived from a Monte Carlo simulation, were compared, such average scores (3.88 ± 0.36 vs. 3.81 ± 0.38 kg CO_2e_/kg) were found to be not statistically different at the 95% confidence level. The same result was obtained when the overall weighted damage scores resulting from the use of the EcoInvent characterization factors of the cut-off (184 ± 20 mPt/kg) or APOS (185 ± 20 mPt/kg) system models were compared.

Finally, Table 9 compares the B2C carbon footprint and overall weighted damage score of 1 kg of low-GI fresh pasta packed in 0.5 PE bags according to the PAS 2050 and ReCiPe 2016 standard methods when doubling the energy needs for fresh pasta manufacture or increasing cooked pasta waste to as much as 20% of cooked fresh pasta. Even in these conditions, there was no statistically significant variation between the aforementioned eco-indicators. In fact, the relative variation of both eco-indicators with respect to the corresponding default score (m_i_) was generally smaller than 1.6% if each parameter X_i_ was increased by +100% with respect to its corresponding default value.

### 4.4. Main Actions to Mitigate the Environmental Impact of Low-GI Fresh Pasta

As shown in Table 8, any mitigation option should aim to reduce the contribution of the field phase firstly and then that of the use phase.

Several studies have shown that organic cultivation of bread wheat gives rise to lower GHG emissions per hectare than conventional cultivation [65]. Regrettably, the lower productivity asks for a greater use of agricultural soil. In this specific case, for the adverse meteorological conditions in the 2020 and 2021 cropping seasons mentioned above, the cultivation of the derived high-amylose line of the bread wheat cultivar Cadenza [3] in two agricultural farms of the Latium region (Table 1) resulted in crop yields quite lower than the average one (7.26 ± 0.48 Mg/ha) for bread wheat, when cultivated in the same agronomic area [63]. Before adopting any soil conservation program, especially because the field tests carried out at Farm 2 had involved direct drilling (Table 1), the main priority would be to improve genetically, the resistance of such a high-amylose cultivar to drought and spring frosts to enhance its crop yield up to the average one mentioned above. In these conditions, the carbon footprint of the high-amylose bread wheat would significantly reduce from 1.17 to 0.28 kg CO_2e_/kg and, consequently, the B2C carbon footprint and overall weighted damage score of the resulting low-GI fresh pasta would decrease from 3.88 to 2.65 kg CO_2e_/kg, and from 184 to 101 mPt/kg (Table 9). Thus, such eco-indicators resulted in about 5.7% and 8.6% higher than those of conventional fresh pasta, this points out the paramount mitigating effect on the impact of the agricultural phase.

To alleviate the impact of the use phase, the conventional gas-fired and electric kitchen appliances are to be replaced with the novel eco-sustainable pasta cooker controlled by an Arduino^®^ microprocessor developed previously [66]. In this way, it would be possible to reduce not only the cooking water from 10 to 3 L per each kg of fresh pasta with no significant change in the main chemico-physical properties of cooked pasta, but also the cooking energy consumption from 1.29 ± 0.04 to 0.58 ± 0.01 kWh/kg [9]. As shown in Table 9, the B2C CF and OWDS of the resulting low-GI fresh pasta would decrease from 3.88 to 2.79 kg CO_2e_/kg, and from 184 to 154 mPt/kg (Table 9).

Also, the energy requirements for preserving fresh pasta in domestic fridges should be minimized. For instance, the general consumer should be encouraged, even with fiscal aids, to replace old refrigerators with new ones of higher energy class. Thus, if the energy classes of all the home fridges in use in Italy were distributed as those acquired in 2018 [50], and if specific advertising campaigns attracted the awareness of the consumer to shorten the storage time of fresh pasta in home refrigerators from the default 30 days to just 10 days, the refrigeration energy need would reduce from 2.46 to 0.55 kWh per kg of fresh pasta. This would reduce the carbon footprint from 3.88 to 3.26 kg CO_2e_ and overall weighted damage score from 184 to 161 mPt/kg (Table 9).

Finally, if all the three mitigation actions mentioned above were simultaneously adopted, it would be possible to cut both eco-indicators by about 60%, that is CF_B2C_ from 3.88 to 1.58 kg CO_2e_/kg, and OWDSB2C from 184 to 68 mPt/kg (Table 9). In the circumstances, the environmental profile of the novel low-GI fresh pasta would be definitively more favorable than that of the conventional fresh pasta (Figure 6), even with a 30% greater score for the land use impact category which was due to a lower flour extraction rate of 54% instead of 73% of input raw grains.

A further remark is conclusively needed to note that the manufacture and consumption of fresh pasta involves greater energy use and GHG emissions than that of dry pasta mainly because of the chilled truck transport of a higher moisture product and its preservation in domestic refrigerators. Thus, the general consumer should be aware that the consumption of RS-enriched dry pasta, as that produced for instance by De Arcangelis et al. [10], instead of this low-GI fresh pasta would give rise to a lower environmental impact, especially if smart energy-saving home appliances are used.

## 5. Conclusions and Future Perspectives

The business-to-business environmental impact of a novel high-amylose bread wheat flour fresh pasta with low in vitro glycemic index was investigated using an LCA approach and compared to that of a conventional fresh pasta made of common wheat flour and water. Not only the carbon footprint according to PAS 2050, but also the global environmental impact using the endpoint ReCiPe 2016 standard method allowed the identification of the same hotspots (i.e., high-amylose bread wheat cultivation and consumer use of fresh pasta as due to cooking and chilled preservation). Nevertheless, the potential consumer requiring low-GI foods should be conscious that the use of the fresh pasta under study would involve about 55% higher carbon footprint and 98% higher overall weighted damage score than conventional fresh pasta, mainly because the smaller high-amylose bread wheat yield per hectare increased land occupation and, consequently, resulted in a greater damage to the human health and ecosystem quality compartments.

By aligning the crop yields obtained using conventional reduced-tillage farming in the cultivation areas tested up to those usually achieved for common wheat in Central Italy, the environmental impact of the low-GI fresh pasta chain examined here tended towards that of the conventional fresh pasta chain within a deviation not greater than 9%. This confirmed the paramount impact of the agricultural phase on the damage to human health and ecosystem quality.

Conversely, the replacement not only of the gas-fired hobs mainly used in Italy, with novel eco-sustainable pasta cookers, but also of the current domestic refrigerators with new models of higher energy class might relieve the damage to resource availability. Of course, such options would positively affect the environmental impact of the conventional fresh pasta chain too.

In conclusion, more sustainable high-amylose bread wheat cultivation and energy- efficient home appliances would significantly relieve the environmental impact of the novel low-GI fresh pasta with improved effects on post-prandial glucose metabolism examined here. Further work is thus needed to confirm the effectiveness of the mitigation actions suggested above.

## Figures and Tables

**Table 1:** Cultural conditions used to grow high-amylose bread wheat in two agricultural farms.

Localization	Farm 1	Farm 2		Unit
Denomination	“Nello Lupori”	“Filzi Massimo”		
Town	Viterbo, I	Anguillara Sabazia, I		
Latitude	42°26′ N	42.060215		
Longitude	12°04′ E	12.258220		
Altitude	310	200		m a.s.l.
Crop year	2019	2020	2021	
Cultivation area	0.3340	1.2	7.3	ha
Grain yield	2395	1500	1000	kg/ha
Seed moisture	12.5	11.0	11.0	% p/p
Storage	in situ	in situ	in situ	
Distance from grain storer to miller	10	12	12	km
Grain drying	no	no	no	
Straw utilization - Left on field: - Incineration: - Other uses	100 0 0	0 0 100	0 0 100	% % %
Below ground residue uses - Left on field: - Incineration: - Other uses	100 0 0	100 0 0	100 0 0	% % %
Seeding density	200	250	250	kg/ha
Diesel fuel consumption	130 ^§^	70 *	70 *	L/ha
Lubricating oil	5	0.2	0.2	L/ha
(NH_4_)_2_HPO_4_ (N: 18%, P_2_O_5_: 46%)	250	200	200	kg/ha
NH_4_NO_3_ (N: 26%)	0	150	150	kg/ha
Urea (N: 46%)	150	150	150	kg/ha
Herbicide	-	0.8	0.8	L/ha
Fungicide	-	1	1	L/ha

^§^ Conventional cultivation; * Sod seeding; a.s.l., above sea level.

**Table 2:** Main results of a few grinding tests using high-amylose bread wheat grains, which were carried out in laboratory- and pilot-scale mills at Grandi Molini Italiani SpA (Venice, Italy).

Input/Output Stream		Lab-Scale Mill	Pilot-Scale Mill	Unit
Processed grain	HABWG	162	7496	kg
Clean grain	GP	n.d.	7200	kg
Grain cleaning waste	TPP + TP	n.d.	296	kg
Tempering water	TW	18	n.d.	%
Wet grain	GPU	n.d.	7417	kg
Flour	HABWF	91	4005	kg
Wheat bran	CR1	48	528	kg
Wheat middlings	CR2		1681	kg
Meal + Germ + Groats	FGT	23	980	kg

n.d.—not determined.

**Table 3:** Fresh pasta packaging: mass of any component of primary, secondary and tertiary packages used.

Packaging Type	Technical Specifications	Unit
*Primary Packaging*	PE bags	
Mass of fresh pasta	0.5	kg
Mass of PE bag	15.7 ± 0.3	g
Length × Width × Height	180 × 65 × 280	mm × mm × mm
Thickness	100	mm
Paper label	1	g
Liquid nitrogen mass	5	g
Liquid carbon dioxide mass	2.5	g
Primary packaging overall mass	0.524	kg
*Secondary Packaging*	Cardboard carton	
No. of primary packages	12	-
Length × Width × Height	400 × 300 × 400	mm × mm × mm
Carton mass	560 ± 1	g
Adhesive label for cartons	2	g
Scotch tape	7	g
Mass of fresh pasta per carton	6.00	kg
Secondary packaging overall mass	6.86	kg
*Tertiary Packaging*	Euro pallet	
Length × Width × Height	1200 × 800 × 144	mm × mm × mm
Pallet mass	22	kg
No. secondary packages	8	-
No. layer per pallet	5	-
Overall height of pallet	2.1414	m
Pallet label	2 × (3.11 ± 0.05)	g
Stretch-and-shrink PE film	684 ± 4	g
Mass of fresh pasta per pallet	240	kg
Tertiary packaging overall mass	297.1	kg

**Table 4:** Logistics of input/output materials with indication of the means of transport used and distance travelled from different production sites to destination ones. All symbols were listed in the Nomenclature section.

Input/Output Materials	From	To	Means of Transport	Load Capacity [Mg]	Distance [km]
HABW seeds	PS	Field	Euro5 L-MRT	3.5–7.5	55
Urea	PS	Field	Euro5 HRT	7.5–16	100
Ammonium nitrate	PS	Field	Euro5 HRT	7.5–16	100
Diammonium phosphate	PS	Field	Euro5 HRT	7.5–16	100
Herbicide	PS	Field	LCV	1.2	25
Fungicide	PS	Field	LCV	1.2	25
Diesel fuel	RDC	Field	LCV	1.2	25
Lubricating oil	RDC	Field	LCV	1.2	25
Straw	Field	CaF	Euro5 HRT	7.5–16	50
HABW grains	Field	WMG	Euro5 HRT	13.9	20
Bread wheat flour type 00	WMG	FPFG	Euro5 HRT	13.9	150
HABW flour	WMG1	FPFG	Euro5 HRT	13.9	60
Kraft paper	FA	PS	Euro5 HAT	16–32	250
25-kg paper bags	PS	WMG	Euro5 HAT	16–32	527
Liquid CO_2_	PS	FPFG	Euro5 HRT	10	95
Liquid N_2_	PS	FPFG	Euro5 HRT	10	95
PE granules	PS	FG	Euro5 HRT	7.5–16	150
PE bags	FG	FPFG	Euro5 HRT	13.9	106
Cartons	PS	FPFG	Euro5 HRT	13.9	65
Raw mat.s for paper label production	PS	FG	Euro5 HRT	7.5–16	250
Adhesive paper labels	FG	FPFG	Euro5 HRT	13.9	65
PE film and scotch tape	FG	FPFG	Euro5 HRT	13.9	100
Raw mat.s for wood pallet production	PS	EPMC	Euro5 HRT	16–32	100
Reusable wood pallet	EPMC/DCE	FPFG/EPMC	Euro5 HRT	13.9	30/100
Broken wood pallet	FPFG/DCE	EPMC	Euro5 HRT	13.9	50
Palletized fresh pasta	FPFG/DCE	DCE/PoS	Euro5 RHRT/RLRT	7.5–16/3.5–7.5	100/30
Detergent (Liquid Cl_2_)	PS	WMG/FPFG	Euro5 LRT	3.5–7.5	112/83
Lubricant oil	PS	WMG/FPFG	Euro5 LRT	3.5–7.5	133/103
Milling by-products	WMG	CaF	Euro5 HRT	13.9	50
Organic or packaging waste	FPFG/WMG/CH	WCC	Euro5 L-MRT	3.5–7.5	50

**Table 5:** Overall Italian waste management scenarios for packaging and organic wastes in 2019, as derived from the fresh pasta processing, distribution, and consumer phases.

Waste Management Scenario	Landfill [%]	Recycling [%]	Incineration [%]	References
Organic wastes	31	51	18	[54,55]
Paper and cardboard wastes	1.6	80.8	7.6	[53]
Wood wastes	34.8	63.1	2.1	[53]
Plastic wastes	7.4	45.6	47.0	[53]
Unsorted MSW	52.6	0.0	47.4	[56]

**Table 6:** Percentage contribution of the different life cycle phases to the business-to-consumer carbon footprint (CF_B2C_) of a functional unit (1 kg) of fresh pasta made of high-amylose bread wheat flour (HABWF) or common wheat flour (CWF) and packed in 0.5-kg PE bags when using the characterization factors extracted from the Cut-off or APOS system model of the Ecoinvent v. 3.8 database.

Fresh Pasta Made of	HABWF				CWF	
EcoInvent v. 3.8 Database	Cut-Off, S		APOS, S		Cut-Off, S	
Life Cycle Phases	[#]	[%]	[#]	[%]	[#]	[%]
Field phase (FIP)	807	20.65	810	21.13	122	4.85
On-field Emissions (FE)	817	20.90	817	21.31	143	5.68
Milling (MI)	82	2.09	81	2.12	60	2.38
Pasta production and modified atmosphere packaging (PMAP)	95	2.43	95	2.47	92	3.65
Packaging material production (PMP)	335	8.59	291	7.59	335	13.37
Transport of packaging, auxiliary materials and wastes (TR_paw_)	96	2.45	96	2.50	82	3.26
Transport of final product (TR_fp_)	36	0.91	35	0.93	36	1.42
Consumer phase (CP)	1554	39.77	1554	40.56	1554	61.96
Cooked fresh pasta waste (CPW)	19	0.48	20	0.53	19	0.75
End-of-life packaging material waste (EoLPM)	68	1.73	33	0.85	68	2.69
*Carbon Footprint* (CF_B2C_)	*3908*	*100.00*	*3832*	*100.00*	*2508*	*100.0*

^#^ [g CO_2e_/kg].

**Table 7:** Environmental profile of 1 kg of low-GI fresh pasta packed in 0.5-kg PE bags, as estimated using the ReCiPe 2016 standard method: Percentage contribution of the different life cycle phases (symbols as in Table 6), and overall score of each mid-point impact category (ICj).

Midpoint Impact Category IC_j_	Life Cycle Phase Contribution (%)									IC_j_ Score			Unit
	FIP + FE	MI	PMAP	PMP	TR_paw_	TR_fp_	CP	CPW	EoLPM	Mean	±	sd	
Global warming (GW_100_)	**42.27**	2.06	2.40	8.48	2.37	0.88	*39.20*	0.57	1.77	3.99 × 10^0^	±	3.83 × 10^−1^	kg CO_2e_
Stratospheric ozone depletion (OD)	**96.44**	0.15	0.17	0.60	0.20	0.07	*2.34*	0.01	0.02	3.49 × 10^−5^	±	1.08 × 10^−5^	kg CFC-11_e_
Ionizing radiation (IR)	13.06	3.75	4.26	*19.81*	0.84	0.19	**57.86**	0.21	0.01	2.82 × 10^−1^	±	6.45 × 10^−3^	kBq ^60^Co_e_
Fine particulate matter formation (PM)	**67.14**	1.26	1.36	7.24	1.45	0.49	*20.90*	0.13	0.04	6.69 × 10^−3^	±	7.99 × 10^−4^	kg PM_2.5e_
O_3_ formation, Human health (OFHH)	**69.16**	1.15	1.22	6.69	2.27	0.79	*18.56*	0.07	0.09	1.18 × 10^−2^	±	1.46 × 10^−3^	kg NO_xe_
O_3_ formation, Terrestrial ecosystems (OFTE)	**81.81**	0.67	0.71	4.00	1.34	0.47	*10.91*	0.04	0.05	2.04 × 10^−2^	±	2.99 × 10^−3^	kg NO_xe_
Terrestrial acidification (TA)	**72.27**	1.16	1.26	5.05	1.04	0.36	*18.71*	0.12	0.03	2.15 × 10^−2^	±	2.77 × 10^−3^	kg SO_2e_
Freshwater eutrophication (FEU)	**71.22**	0.71	0.83	11.54	0.66	0.25	*12.83*	0.32	1.65	2.75 × 10^−3^	±	3.48 × 10^−4^	kg P_e_
Marine eutrophication (MEU)	**90.85**	0.05	0.08	2.30	0.03	0.02	*2.74*	3.65	0.29	3.41 × 10^−3^	±	1.39 × 10^−3^	kg N_e_
Terrestrial ecotoxicity (TECO)	**39.49**	0.55	0.90	8.32	12.15	3.44	*33.88*	-0.02	1.28	1.13 × 10^1^	±	8.31 × 10^−1^	kg 1,4-DCB
Freshwater ecotoxicity (FECO)	*25.37*	0.55	0.74	7.01	1.39	0.65	**61.76**	0.03	2.51	1.71 × 10^−1^	±	7.64 × 10^−3^	kg 1,4-DCB
Marine ecotoxicity (MECO)	*26.10*	0.59	0.79	7.25	1.78	0.73	**59.95**	0.04	2.79	2.20 × 10^−1^	±	1.01 × 10^−2^	kg 1,4-DCB
Human carcinogenic toxicity (HCT)	*32.18*	1.73	2.14	14.09	3.74	1.20	**44.73**	-0.10	0.30	1.19 × 10^−1^	±	6.90 × 10^−3^	kg 1,4-DCB
Human non-carcinogenic toxicity (HNCT)	*36.19*	1.38	1.66	13.31	3.23	0.70	**41.44**	0.23	1.87	2.37 × 10^0^	±	1.53 × 10^−1^	kg 1,4-DCB
Land use (LU)	**97.94**	0.02	0.02	1.59	0.05	0.01	0.37	0.01	0.00	9.00 × 10^0^	±	1.59 × 10^0^	m^2^ yr crop_e_
Mineral resource scarcity (MRS)	**70.32**	0.45	0.60	6.68	1.69	0.53	*19.75*	-0.08	0.04	1.36 × 10^−2^	±	1.71 × 10^−3^	kg Cu_e_
Fossil resource scarcity (FRS)	*32.20*	2.36	2.62	12.18	3.00	0.98	**46.60**	0.04	0.02	1.07 × 10^0^	±	6.10 × 10^−2^	kg oil_e_
Water consumption (WS)	**67.32**	1.50	1.85	6.23	0.19	0.05	*22.78*	0.07	0.02	1.01 × 10^−1^	±	1.21 × 10^−2^	m^3^

The percentage contribution in bold or Italics type represents the primary or secondary hotspot of each IC_j_, respectively.

**Table 8:** Endpoint characterization of the environmental profile of 1 kg of fresh pasta packed in 0.5-kg PE bags according to the ReCiPe 2016 standard method: percentage contribution of the different life cycle stages (symbols as in Table 6), single (SS_z_) and weighted damage scores (WDS_z_) of each damage category (DC_z_), and overall weighted score (OWDS).

Damage Category (DCz)	Life Cycle Phase Contribution (%)									SS_z_			Unit	WDS_z_
	FIP + FE	MI	PMAP	PMP	TR_paw_	TR_fp_	CP	CPW	EoLPM	Mean	±	sd		mPt/kg
Human health (HH)	**54.82**	1.58	1.80	8.18	1.95	0.67	*29.87*	0.30	0.84	9.27 × 10^−6^	±	9.32 × 10^−7^	^¥^	*155 ± 16*
Ecosystem quality (EQ)	**89.48**	0.33	0.38	2.81	0.41	0.14	*6.14*	0.08	0.23	1.00 × 10^−7^	±	1.60 × 10^−8^	^§^	*27 ± 3*
Resource availability (RA)	*35.21*	2.08	2.31	11.98	3.78	1.22	**43.41**	-0.01	0.02	3.62 × 10^−1^	±	2.25 × 10^−2^	^#^	*2.6 ± 0.2*
***OWDS***	**59.74**	1.40	1.59	7.43	1.75	0.59	*26.50*	0.26	0.74					***184 ± 20***

^¥^ DALY/kg; ^§^ Species yr/kg; ^#^ US$2013/kg. The percentage contribution in bold or Italics type represents the first or second hotspot, respectively.

**Table 9:** Effect of the sensitivity analysis and some mitigation actions on the B2C carbon footprint and overall weighted damage score of 1 kg of low-GI fresh pasta according to the PAS 2050 and ReCiPe 2016 standard methods and corresponding percentage variation ratio, m_CF,i_ or m_OWDS,i_, towards different percentage variation of each sensitivity or mitigation parameter X_i_ accounted for.

Case No.	Sensitivity or Mitigation Parameter X_i_	Value	Unit	Ref.	CF_B2C_ [kg CO_2e_/kg]	m_CF,i_ [%]	OWDS_B2C_ [mPt/kg]	m_OWDS,i_ [%]
1	Reference Value	-	-	This work	3.88 ± 0.37	-	184 ± 20	-
2	Higher electric and thermal energy needs	400 40	kWh/Mg kWh/Mg	This work This work	3.93 ± 0.36 3.88 ± 0.37	1.3 0.0	187 ± 21 186 ± 21	1.6 1.1
3	Higher cooked pasta waste	20	%	[57]	4.01 ± 0.35	0.4	187 ± 20	0.2
4	Higher HABW yield	7.26	Mg/ha	[63]	2.65 ± 0.07	-8.3	101 ± 2	-11.7
5	Smaller cooking energy needs	0.58 ± 0.01	kWh/kg	[66]	2.79 ± 0.37	51.0	154 ± 20	29.6
6	Smaller refrigeration energy needs	0.55	kWh/kg	This work	3.26 ± 0.07	20.6	161 ± 20	16.1
7	Simultaneous options no. 4, 5, and 6	7.26 0.58 0.55	Mg/ha kWh/kg kWh/kg	This work	1.58 ± 0.06	-	68 ± 2	-

## Data Availability

Data is contained within the article.

## Supplementary Materials

The following supporting information can be downloaded at: https://www.mdpi.com/article/10.3390/foods11203199/s1. Table S1: description of the six product stages of the fresh pasta life cycle network; Table S2: input/output data for the conventional production of HABW; Table S3: inventory of the HABW cultivation phase; Table S4: material balance of the HABW milling step in the pilot-scale mill; Table S5: material balances of the HABW milling step referred to 1 ha; Table S6: inventory of 25-kg paper bag production; Table S7: inventory of HABWF production; Table S8: material balances of the HABW fresh pasta making, pasteurization, and pre-drying processes; Table S9: inventory of the HABWF fresh pasta production; Table S10: waste fractions of processing and packaging materials; Table S11: Material balance of the HABW fresh pasta packaging process; Table S12: inventory of the PE bag production; Table S13; inventory of the paper label production; Table S14: inventory of the carton production; Table S15: inventory of the scotch tape production; Table S16: inventory of the EPAL wood pallet production; Table S17: inventory of the shrink and shrank PE film production; Table S18: assembly of the fresh pasta primary packaging; Table S19: assembly of the fresh pasta secondary packaging Table S20: assembly of the fresh pasta tertiary packaging; Table S21: assembly of the fresh pasta primary and secondary packages; Table S22: assembly of the fresh pasta primary, secondary and tertiary packages; Table S23: assembly of fresh pasta; Table S24: inventory of the use phase; Table S25: waste scenario for plastic packaging wastes; Table S26: waste scenario for cardboard and paper packaging wastes; Table S27: waste scenario of wooden pallet wastes; Table S28: end-of-life of primary packaging material wastes; Table S29: end-of-life of secondary packaging material wastes; Table S30: end-of-life of tertiary packaging material wastes; Table S31: end-of-life scenario for the primary, secondary and tertiary packaging material wastes, excluding pallets; Table S32: reuse phase of the wooden pallet; Table S33: life cycle of HABWF fresh pasta; Table S34: life cycle of the primary, secondary, and tertiary packaging materials; Table S35: life cycle of the wooden pallet; Table S36: inventory of bread wheat cultivation in Central Italy; Table S37: inventory of common wheat milling; Table S38: inventory of BWF fresh pasta production.

## Nomenclature

APOS	Allocation at the point of substitution
a_W_	Water activity [dimensionless]
B2B	Business-to-business
B2C	Business-to-consumers
CA	Cartons [kg]
CaF	Cattle farm
CF_B2B_	Business-to-business carbon footprint of a functional unit [kg CO_2e_/kg]
CF_B2C_	Business-to-consumer carbon footprint of a functional unit [kg CO_2e_/kg]
CFC	Trichlorofluoromethane
CH	Consumer house
CO_2e_	Carbon dioxide equivalent
CO_2L_	Liquid carbon dioxide [kg]
CP	Consumer phase
CPW	Cooked fresh pasta waste
CR1	Wheat bran [kg]
CR2	Wheat middlings [kg]
CW	Cooking water
CWF	Common wheat flour
DALY	Disability-adjusted life years
DCB	Dichlorobenzene
DCE	Distribution center
DCz	Generic z-th damage category
DF	Diesel fuel
EC	Carton label [kg]
EE	Electric energy
EoL	End of life
EoLPM	End of life of packaging material wastes.
EP	Wooden pallet label [kg]
EPAL	European Pallet Association
EPD	Environmental Product Declaration
EPMC	Euro pallet managing center
EQ	Ecosystem Quality
ES	PE bag label [kg]
FA	Forest area
FE	On-field emissions
FECO	Freshwater ecotoxicity
Fert	Fertilizers
FEU	Freshwater eutrophication
FG	Generic factory gate
FGT	Meal, germ and groats [kg]
F_i,j_	Characterization factor of the j-th impact category for the generic i-th activity
FIP	Field phase
FP	Wooden pallet wrap film [kg]
FPFG	Fresh pasta factory gate
FRS	Fossil resource scarcity
GHG	Greenhouse gas
GI	Glycemic index
GP	Cleaned grains [kg]
GPP	Pre-cleaned grains [kg]
GPU	Wet cleaned grains [kg]
GW_100a_	100-year time-horizon global warming
HA	High-amylose
HABWG	High-amylose bread wheat grains [kg]
HABWF	High-amylose bread wheat flour [kg]
HABWFS	High-amylose bread wheat flour packed in paper bags [kg]
HAT	Euro5 Heavy articulated truck
HCT	Human carcinogenic toxicity
HH	Human Health
HNCT	Human non-carcinogenic toxicity
HRT	Heavy rigid truck
I	Pasta dough [kg]
IC_j_	Generic j-th impact category
IPCC	Intergovernmental Panel on Climate Change
IR	Ionizing radiation
ISS	Grinding waste (impurities, stones, weed seeds, straw, etc.) [kg]
LCA	Life-cycle assessment
LCV	Light Commercial Vehicle
L-MRT	Light-medium rigid truck
LRT	Light rigid truck
LU	Land use
LubO	Lubricating oil
MAP	Modified atmosphere packaging
MECO	Marine ecotoxicity
MEU	Marine eutrophication
m_i_	Relative variation of the generic ecoindicator as referred to the relative variation of each independent parameter X_i_, each variation being referred to the corresponding reference value [dimensionless]
MI	Milling phase
MRS	Mineral resource scarcity
MSW	Municipal solid waste
N_2L_	Liquid nitrogen [kg]
OD	Stratospheric ozone depletion
OFHH	Ozone formation, Human health
OFTE	Ozone formation, Terrestrial ecosystems
OW	Organic waste [kg]
OWDS	Overall weighted damage score [mPt]
PAL	Wooden pallet [kg]
PAS	Publicly Available Specification
PCW	Paper and cardboard waste [kg]
PE	Polyethylene or PE bag [kg]
Pest	Pesticides
PFC	Fresh pasta in secondary packages [kg]
PFP	Fresh pasta in tertiary packages [kg]
PFS	Fresh pasta in primary packages [kg]
PMP	Packaging material production
PMAP	Pasta production and modified atmosphere packaging
PM	Fine particulate matter formation
PM_2.5_	Particulate matter with aerodynamic diameter not greater than 2.5 μm
PoS	Point of sale
PP	Pasteurized fresh pasta ready to be packaged [kg]
PPU	Pasteurized wet fresh pasta [kg]
PS	Production site
PTU	Extruded fresh pasta [kg]
PW	Plastic waste [kg]
Q	Thermal energy
RA	Resource availability
RDC	Regional distribution center
RHRT	Refrigerated heavy rigid truck
RLRT	Refrigerated light rigid truck
RS	Resistant starch
SAF	Paper bags [kg]
SC	Carton scotch tape [kg]
SCA	Carton waste [kg]
SEC	Carton label waste [kg]
SEP	Wooden pallet label waste [kg]
SES	Bag label waste [kg]
SFP	Wooden pallet wrap film waste [kg]
SI	Dough discarded during kneading [kg]
SPAL	Wooden pallet waste [kg]
SPE	PE bag waste [kg]
SPP	Fresh pasta waste [kg]
SSAF	Paper bag waste [kg]
SSC	Scotch tape waste [kg]
SSz	Single damage score of the z-th damage category
TA	Terrestrial acidification
TECO	Terrestrial ecotoxicity
TP	Cleaning dockage [kg]
TPP	Pre-cleaning dockage [kg]
TR	Transportation
TRfp	Transport of final product
TRpaw	Transport of packaging and auxiliary materials, and wastes
TW	Tempering water [kg]
VAT	Water vapor removed during pasta pre-drying [kg]
VP	Saturated steam needed to pasteurize fresh pasta [kg]
VT	Water evaporated during dough extrusion [kg]
WCC	Waste collection center
WDS_z_	Weighted damage score of the z-th damage category [mPt]
WE	Water evaporated during wheat milling [kg]
WhS	Wheat seeds
WFP	Wheat feed pellets [kg]
WI	Water used to prepare the dough [kg]
WMG	Wheat mill gate
WS	Water consumption
WW	Wood waste [kg]
X_i_	Generic i-th independent parameter
x_W_	Moisture content [g/g1]
*Greek Symbols*	
ΔCF_B2C_	Relative variation of CF_B2C_ with respect to the reference value (=CF_B2C_ − CF_B2C,R_) [kg CO_2e_/kg]
ΔOWDS	Relative variation of OWDS with respect to the reference value (=OWDS − OWDS_R_) [mPt/kg]
ΔX_i_	Relative variation of each independent parameter X_i_ with respect to the corresponding reference value (X_iR_)
Ψ_i,j_	Entity of the i-th activity on the j-th impact category [kg, J or Mg km]
*Subscript*	
R	Reference value
