# Supplementary material for: Environmental Profile of a Novel High-Amylose Bread Wheat Fresh Pasta with Low Glycemic Index
Source: Foods. 2022 Oct 13;11(20):3199. doi: 10.3390/foods11203199 (PMC9601865; doi:10.3390/foods11203199)
Supplement: Supplementary file 1 [file foods-11-03199-s001.zip › foods-1929422-supplementary.pdf]

SUPPLEMENTARY MATERIAL

Table S1. Description of the six product stages involved in the fresh pasta life cycle network according to the Simapro software used in this work.

Product stage	Name	Main output	Unit	Reference Table
Process	High-amylose soft wheat cultivation	HABW grain	kg	Table S3
Process	25-kg paper bag production	SAF	kg	Table S5
Process	High-amylose bread wheat milling	HABW flour	kg	Table S6
Process	High-amylose soft bread pasta production	Fresh pasta	kg	Table S7
Process	PE bag production	PE bags	kg	Table S9
Process	Adhesive paper label production	Paper labels	kg	Table S10
Process	Carton production	Cartons	kg	Table S11
Process	Scotch tape production	Scotch tape	kg	Table S12
Process	EPAL wood pallet production	Wood pallet	kg	Table S13
Process	Shrink & shrank PE film production	PE film	kg	Table S14
Assembly	Assembly of the primary packaging for fresh pasta	Ass 1pack	p	Table S15
Assembly	Assembly of the secondary packaging for fresh pasta	Ass 2pack	p	Table S16
Assembly	Assembly of the tertiary packaging for fresh pasta	Ass 3pack	p	Table S17
Assembly	Assembly of the primary and secondary packages for fresh pasta	Ass 1-2pack	p	Table S18
Assembly	Assembly of the primary, secondary and tertiary packages for fresh pasta	Ass 1-3pack	p	Table S19
Assembly	Assembly of fresh pasta	Ass pasta	p	Table S20
Processes	Use phase	Cooked fresh pasta	kg	Table S21
Waste scenario	Waste scenario of plastic packaging wastes	PW	kg	Table S22
Waste scenario	Waste scenario of paper and cardboard packaging wastes	PCW	kg	Table S23
Waste scenario	Waste scenario of wooden pallet wastes	PALW	kg	Table S24
End of life scenario	End of life scenario of the primary packaging material wastes	EoL-1pack	-	Table S25
End of life scenario	End of life scenario of the secondary packaging material wastes	EoL-2pack	-	Table S26
End of life scenario	End of life of the tertiary packaging wastes	EoL-3pack	-	Table S27
End of life scenario	End of life scenario of the I, II, and III packaging material wastes	EoL-1-3pack	-	Table S28
Reuse	Reuse of the wooden pallet	Pallet reuse	p	Table S29
Life cycle	Life cycle of fresh pasta	LC-FP	p	Table S30
Life cycle	Life cycle of the primary, secondary and tertiary packaging materials	LC1-3pack	p	Table S31
Life cycle	Life cycle of the wooden pallet	LC-3pack	p	Table S32

Table S2 Input/output data for the conventional production of high-amylose bread wheat at the Massimo Filzi farm (Anguillara Sabazia, Italy), as referred to a nominal land area of 1 ha. All emission factors were extracted from the recently updated IPCC Guidelines [39].

**Inpu/Output Parameter**	**Unit**	**Amount**
Nominal non-irrigated land used (A_C)	ha	1.0
Land used to cultivate HASW	%	100
Set-aside land	%	0
HASW seed density	kg · ha^{-1}	250
Distance travelled by seeds (Viterbo-Anguillara Sabazia, Italy)	km	62
Input		
Overall synthetic nitrogen supplied (F_S)	kg N a^{-1}	144
Output		
Crop yield	Mg · ha^{-1} · a^{-1}	1.5
Average grain moisture	g · kg^{-1}	110
Dry grain yield	kg_{DM} · ha^{-1} · a^{-1}	1335
Above ground residues (AG_{DM})	Mg_{DM} · ha^{-1} · a^{-1}	2.55
N content of above-ground residues	kg N · $(kg_{DM})^{-1}$	0.006
- Percentage of straw left in the field	%	0
- Percentage of straw incinerated	%	0
- Percentage of straw baling	%	100
Above ground biomass (AGR)	(kg_{DM}) ha^{-1} a^{-1}	2549
Below ground-to-above ground biomass ratio (R_{BG-BIO})	kg · kg^{-1}	0.23
Below ground residues (BG_{DM})	kg_{DM} · ha^{-1} · a^{-1}	893
N content of below-ground residues	kg N · $(kg_{DM})^{-1}$	0.009
Nitrogen in crop residues (F_{CR})	kg N · ha^{-1} · a^{-1}	8.04
N emissions as NH_3- ed NO_x ($Frac_{GASF}$)	kg NH_3-N+NO_x-N · $(kg\ N)^{-1}$	0.11 (0.02-0.33)
N leaching off ($Frac_{LEACH}$)	kg N · $(kg\ N)^{-1}$	0.24 (0.01-0.73)
N_2O-N emissions per unit mass of N fertilizer (EF_1)	kg N_2O-N · $(kg\ N)^{-1}$	0.01 (0.001-0.018)
N_2O-N emissions per unit mass of NH_3 and NO_X emitted (EF_4)	kg N_2O-N · $(kg\ NH_3\text{-}N+kg\ NO_x\text{-}N\ emitted)^{-1}$	0.01 (0.002-0.018)
N2O-N emissions per unit mass of N leached off (EF_5)	kg N_2O-N · $(kg\ N\ leached\ off)^{-1}$	0.011 (0.0-0.020)
Direct N_2O emissions [=(F_S+F_{CR}) · EF_1 · 44/28]	kg N_2O ha^{-1} yr^{-1}	2.389
Indirect N_2O emissions from NH_3 e NO_x [=F_S $Frac_{GASF}$ · EF_4 · 44/28]	kg N_2O ha^{-1} yr^{-1}	0.249
Indirect N_2O emissions from N leaching off [=(F_S+F_{CR}) $Frac_{LEACH}$ · EF_5 · 44/28]	kg N_2O · $ha^{-1\cdot}$ yr^{-1}	0.631
Direct and indirect N_2O emissions	kg N_2O · $ha^{-1\cdot}$ yr^{-1}	3.269
Urea emission factor (EF_{UREA})	kg C/kg Urea	0.2
Reduction (η)	kg C/kg Urea	0.5
CO_2 emissions from urea (F_{CO2}= Urea A_C EF_{Urea} η)	kg CO_{2e} ha^{-1} yr^{-1}	55.00
NO emissions [40]	kg N ha^{-1} yr^{-1}	2.142
Nitrate emissions in water	kg NO_3-N ha^{-1} yr^{-1}	36.49
P emissions in water [0.05 kg P/ kg P applied]	kg P ha^{-1} yr^{1}	2.01

Table S3. Inventory associated with the high-amylose bread wheat cultivation phase.

Products

Outputs to technosphere: Products and co-products	Amount	Unit	Quantity	Allocatio	Waste type	Category	Comment
FP-1 High Amylose Wheat Grain	CropYield = 1.5E3	kg	Mass	92.5 %	Biopolymer	A...\Transformation	
STRAW	STRAW/SSSTRAW = 2.92	ton	Mass	7.5 %	Biopolymer	A...\Transformation	
Add							

Outputs to technosphere: Avoided products	Amount	Unit	Distribution	SD2 or 2SD	Min	Max	Comment
Add							

Inputs

Inputs from nature	Sub-compartment	Amount	Unit	Distribution	SD2 or 2SD	Min	Max	Comment
Occupation, annual crop, IT		AREA = 1	ha a					
Add								

Inputs from technosphere: materials/fuels	Amount	Unit	Distrit	SD2 (	Min	Max	Comment
Urea {RER}| urea production | Cut-off, S	UREA*AREA = 150	kg					See Table 1
Diammonium phosphate {RER}| diammonium phosphate production | Cut-off, S	FOSFBIAM*AREA = 200	kg					"
Ammonium nitrate {RER}| ammonium nitrate production | Cut-off, S	NITRAMM*AREA = 150	kg					"
Pesticide, unspecified {RER}| production | Cut-off, U	1.8*AREA = 1.8	kg					Fungicide (1 L/ha) + Herbicide (0.8 L/ha)
Diesel {Europe without Switzerland}| market for | Cut-off, S	70*AREA = 70	kg					See Table 1
Lubricating oil {RER}| market for lubricating oil | Cut-off, S	0.2*AREA = 0.2	kg					"
Wheat seed, for sowing {GLO}| production | Cut-off, S	Seed*AREA = 250	kg					"
Add							

Inputs from technosphere: electricity/heat	Amount	Unit	Distributior	SD2 (	Min	Ma	Comment
Transport, freight, lorry 3.5-7.5 metric ton, EURO5 {RoW}| transport, freight, lorry 3.5-7.5 metric t	Seed*AREA*55 = 1.38E4	kgkm					Transport seedsi VT-Anguillara Sabazia: 55 km
Transport, freight, lorry 7.5-16 metric ton, EURO5 {RER}| transport, freight, lorry 7.5-16 metric tor	(UREA+FOSFBIAM+NITRAMM)*/	kgkm					TR fertilizers Varie-Anguillara Sabazia: 100 km
Transport, freight, light commercial vehicle {Europe without Switzerland}| market for transport, 1	(1.8+70+0.2)*AREA*25 = 1.8E3	kgkm					TR pest+diesel fuel+Lubr oil Varie-Angu. Sab.: 25 km
Transport, freight, lorry 7.5-16 metric ton, EURO5 {RER}| transport, freight, lorry 7.5-16 metric tor	STRAW/SSSTRAW*50 = 146	tkm					TR Paglia Ang Sab-Varie: 50 km
Add							

Outputs

Emissions to air	Sub-compartment	Amount	Unit	Distrib	SD2 or 2	Min	Max	Comment
Dinitrogen monoxide	low. pop., long-ter	FN2OTOT = 3.27	kg					
Nitrogen monoxide, IT	low. pop., long-ter	NOA = 4.59	kg					
Carbon dioxide, fossil	low. pop., long-ter	CO2U = 55	kg					
Add								

Emissions to water	Sub-compartment	Amount	Unit	Distributio	SD2 or 2SI	Min	Max	Comment
Nitrate, IT	groundwater, long	NITRL = 36.5	kg					
Phosphorus, IT	groundwater, long	PLEACH = 2.01	kg					
[illegible]								

Table S4 Total and partial material balances of the high-amylose bread wheat milling step in the pilot-scale mill with the pre-cleaning, cleaning, and grinding yields.

**Step**			**Pre-cleaning**			**Cleaning**		**Tempering**			**Grinding**						**Pelletizing**	
**Stream**	**HASWG**		**ISS**	**TPP**	**GPP**	**TP**	**GP**	**TW**	**GPU**		**CR1**	**CR2**	**FGT**	**WE**	**HAWF**		**WFP**	
	kg	% p/p	kg	kg	kg	kg	kg	kg	kg	% p/p	kg	kg	kg	kg	kg	% p/p	kg	% p/p
Water	824.6	11	0.41	7.83	816.3	41.7	774.6	375.0	1149.6	**15.5**	68.0	216.5	126.2	223.0	515.9	12.88	460.29	12.6
Dry matter	6671.4	89	3.34	63.38	6604.7	337.4	6267.4	0.0	6267.4	84.5	460.0	1464.5	853.8	0.0	3489.1	87.12	3178.97	87.4
Total	***7496.0***	***100***	***3.75***	***71.21***	***7421.0***	***379.1***	***7042.0***	***375.0***	***7417.0***	***100.0***	***528.0***	***1681.0***	***980.0***	***223.0***	***4005.0***	***100.0***	***3639.27***	***100.0***
Yield (%)			*0.05*	*0.95*	*99.00*	*5.11*	*94.89*	*5*	*105*		*7*	*23*	*13*	*3*	*54*		*49*	

Table S5. Total and partial material balances of the milling step of the HABW grains harvested from a nominal fertilized soil area of 1 ha. All symbols were listed in the Nomenclature section.

**Step**			**Pre-cleaning**			**Cleaning**		**Tempering**			**Grinding**						**Pelletizing**		**Packaging**			
**Stream**	**HABWG**		**ISS**	**TPP**	**GPP**	**TP**	**GP**	**TW**	**GPU**		**CR1**	**CR2**	**FGT**	**WE**	**HABWF**		**WFP**			**SAF**	**SSAF**	**HABWFS**
	kg	% p/p	kg	kg	kg	kg	kg	kg	kg	% p/p	kg	kg	kg	kg	kg	% p/p	kg	% p/p	no.	kg	kg	kg
Water	165	11	0.08	1.57	163.4	8.3	155.0	75	230.0	**15.5**	13.6	43.3	25.3	44.6	103.2	12.88	92.11	12.6				
Dry matter	1335	89	0.67	12.68	1321.7	67.5	1254.1	0	1254.1	84.5	92.0	293.1	170.8	0	698.2	87.12	636.13	87.4				
Total	*1500*	*100*	*0.75*	*14.25*	*1485.0*	*75.9*	*1409.1*	*75*	*1484.19*	*100.0*	*105.7*	*336.4*	*196.1*	*44.6*	*801.4*	*100.00*	*728.24*	*100.0*	*32.1*	*3.72*	*0.04*	*805.11*

Table S6. Inventory associated with the production of 25-kg paper bags.

Products

Outputs to technosphere: Products and co-products	Amount	Unit	Quantity	Allocatio	Waste type	Category	Comment
FP-1 bis paper bags x flour	1	kg	Mass	100 %	Paper	P...\Transformation	

Add

Outputs to technosphere: Avoided products	Amount	Unit	Distribution	SD2 or 2SD	Min	Max	Comment

Add

Inputs

Inputs from nature	Sub-compartment	Amount	Unit	Distribution	SD2 or 2SD	Min	Max	Comment

Add

Inputs from technosphere: materials/fuels	Amount	Unit	Distrit	SD2 (	Min	Max	Comment
Paper sack {RER}| market for paper sack | Cut-off, S	1	kg	Unde				

Add

Inputs from technosphere: electricity/heat	Amount	Unit	Distribu	SD2	Mi	Ma	Comment
Transport, freight, lorry 16-32 metric ton, EURO5 {RER}| transport, freight, lorry 16-32 metric ton,	1*250 = 250	kgkm					TR Kraft paper raw material from forest area : 250 km

Table S7. Inventory associated with the high-amylose bread wheat flour production step.

Products

Outputs to technosphere: Products and co-products	Amount	Unit	Quantity	Allocatio	Waste type	Category	Comment
FP- 2 High Amylose Wheat Flour, at industrial mill	HAWG*YHAWF = 801	kg	Mass	84 %	Biopolymer	Food\Cereal produ	Economic allocation factors (UNAFTA, 2018)
FP-Pelletized Middlings	HAWG*YWFP = 728	kg	Mass	16 %	Biopolymer	Food\Cereal produ	
FP-ISS	HAWG*YISS = 0.75	kg	Mass	0 %	Others	Food\Cereal produ	Impurities
Add							

Outputs to technosphere: Avoided products	Amount	Unit	Distribution	SD2 or 2SD	Min	Max	Comment
Add							

Inputs

Inputs from nature	Sub-compartment	Amount	Unit	Distribution	SD2 or 2SD	Min	Max	Comment
Add								

Inputs from technosphere: materials/fuels	Amount	Unit	Distrit	SD2 ‹	Min	Max	Comment
FP-1 High Amylose Wheat Grain	HAWG = 1.5E3	kg					MP
Tap water {Europe without Switzerland}| tap water production, underground water without treatment |	HAWG*YMO = 75	kg					
FP-1 bis paper bags x flour	NSF*MSF*(1+SPSAF) = 3.72	kg					
Chlorine, liquid {RER}| chlorine production, liquid | Cut-off, S	DETER = 0.00255	kg					
Lubricating oil {RER}| market for lubricating oil | Cut-off, S	OLUBR = 0.00315	kg					

Inputs from technosphere: electricity/heat	Amount	Unit	Dis	SI	M	Ma	Comment
Electricity, medium voltage {IT}| electricity voltage transformation from high to medium voltage | Cut-off, S	147*HAWG/1000 = 221	kWh					147 kWh/Mg
Transport, freight, lorry 7.5-16 metric ton, EURO5 {RER}| transport, freight, lorry 7.5-16 metric ton, EURO5 | Cut-c	HAWG*20 = 3E4	kgkm					TR HAWF grains Anguillara Sabazia-Bracciano: 20
Transport, freight, lorry 16-32 metric ton, EURO5 {RER}| transport, freight, lorry 16-32 metric ton, EURO5 | Cut-o	NSF*MSF*(1+SSAF)*527 = 2.01E3	kgkm					TR paper bags Rho (MI)-Spoleto (PG): 527 km
Transport, freight, lorry 3.5-7.5 metric ton, EURO5 {RER}| transport, freight, lorry 3.5-7.5 metric ton, EURO5 | Cut-	(HAWG*YISS+SSAF)*50 = 39.3	kgkm					TR ISS+paper bag waste: 50 km
Transport, freight, lorry 3.5-7.5 metric ton, EURO5 {RER}| transport, freight, lorry 3.5-7.5 metric ton, EURO5 | Cut-	DETER/1000*112 = 0.000286	kgkm					TR Detergent- Nera Montoro (TR) -Bracciano: 112
Transport, freight, lorry 3.5-7.5 metric ton, EURO5 {RER}| transport, freight, lorry 3.5-7.5 metric ton, EURO5 | Cut-	OLUBR/1000*133 = 0.000419	kgkm					TR Lubrificating oil -Terni-Bracciano: 133 km
Transport, freight, lorry 7.5-16 metric ton, EURO5 {RER}| transport, freight, lorry 7.5-16 metric ton, EURO5 | Cut-c	HAWG*YWFP/1000*50 = 36.4	tkm					TR Milling by-products: MFG-CF: 50 km

Emissions to air	Sub-compartment	Amount	Unit	Distrib	SD2 or	Min	Max	Comment
Water	low. pop.	44.6	kg	Undef				see Table 3
Add								

Emissions to water	Sub-compartment	Amount	Unit	Distributio	SD2 or 2SI	Min	Max	Comment
Add								

Emissions to soil	Sub-compartment	Amount	Unit	Distribution	SD2 or 2SD	Min	Max	Comment
Add								

Final waste flows	Sub-compartment	Amount	Unit	Distribution	SD2 or 2SD	Min	Max	Comment
Add								

Non material emissions	Sub-compartment	Amount	Unit	Distribution	SD2 or 2SD	Min	Max	Comment
Add								

Social issues	Sub-compartment	Amount	Unit	Distribution	SD2 or 2SD	Min	Max	Comment
Add								

Economic issues	Sub-compartment	Amount	Unit	Distribution	SD2 or 2SD	Min	Max	Comment
Add								

Outputs to technosphere: Waste and emissions to treatment	Amount	Unit	Distribu	SD2 o	Min	Max	Comment
Inert waste, for final disposal {RoW}| treatment of inert waste, inert material landfill | Cut-off, S	HAWG*YISS = 0.75	kg					
Paper (waste treatment) {GLO}| recycling of paper | Cut-off, S	SSAF*.808 = 0.0298	kg					
Waste paperboard {RoW}| treatment of, sanitary landfill | Cut-off, S	SSAF*.116 = 0.00428	kg					
Waste paperboard {Europe without Switzerland}| treatment of waste paperboard, municipal incineration | Cut-off, S	SSAF*.076 = 0.0028	kg					

Table S8. Total and partial material balances of the fresh pasta making, pasteurization, and pre-drying processes process using the HABW flour obtained by milling the HABW grains harvested from a nominal fertilized soil area of 1 ha (cf. Table S4). All symbols were listed in the Nomenclature section.

**Step**	**Kneading**								**Extrusion**				**Pasteurization**				**Pre-drying/Cooling**			
**Stream**	**HABWF**		**WI**		**SI**		**I**		**VT**		**PTU**		**VP**		**PPU**		**VAT**		**PP**	
	kg	%	kg	%	kg	%	kg	%	kg	%	kg	%	kg	%	kg	%	kg	%	kg	%
Water	103.2	12.88	296.5	100.0	1.6	36.41	398.2	36.4	85.7	100	312.4	31.0	50.4	100.0	362.8	34.29	143.2	100.00	219.6	24.0
Dry matter	698.2	87.12	0.0	0.0	2.8	63.59	695.4	63.6	0.0	0	695.4	69.0	0.0	0.0	695.4	65.71	0.0	0.00	695.4	76.0
Total	*801.4*	*100.0*	*296.5*	*100.0*	*4.4*	*100.0*	*1093.6*	*100.0*	*85.7*	*100*	*1007.9*	*100.0*	50.4	100.0	1058.2	100.0	143.2	100.00	915.0	100.0

Table S9. Inventory associated with the high-amylose bread wheat fresh pasta production step.

Products

Outputs to technosphere: Products and co-products	Amount	Unit	Quantity	Allocatio	Waste type	Category	Comment
FP- 3 Fresh Pasta from HAWF, at packaging	PFPACK = 901	kg	Mass	100 %	Compost	Food\Cereal produ	
FP-SI-SPP	ORGW = 18.1	kg	Mass	0 %	Biopolymer	Food\Cereal produ	Dough (SI) & pasteurized fresh pasta (SPP) wastes (Tables 4&6)

Add

Outputs to technosphere: Avoided products	Amount	Unit	Distribution	SD2 or 2SD	Min	Max	Comment

Add

Inputs

Inputs from nature	Sub-compartment	Amount	Unit	Distribution	SD2 or 2SD	Min	Max	Comment

Add

Inputs from technosphere: materials/fuels	Amount	Unit	Distrit	SD2 (	Min	Max	Comment
FP- 2 High Amylose Wheat Flour, at industrial mill	HAWF = 801	kg					
Tap water {Europe without Switzerland}| market for | Cut-off, S	HAWF*WFR = 297	kg					Water for dough preparation
Water, deionised {Europe without Switzerland}| water production, deionised | Cut-off, S	50.4	kg	Unde				To obtan steam x fresh pasta pasteurization
Chlorine, liquid {GLO}| production | Cut-off, S	DETER = 0.0141	kg					
Lubricating oil {RER}| market for lubricating oil | Cut-off, S	OLUBR = 0.0266	kg					

Add

Inputs from technosphere: electricity/heat	Amount	Unit	Distrib	SD2	Mi	Ma	Comment
Heat, central or small-scale, natural gas {Europe without Switzerland}| heat production, natu	PFPACK/1000*20 = 18	kWh					Thermal energy 20 kWh/Mg fresh pasta
Electricity, medium voltage {IT}| electricity voltage transformation from high to medium vol	PFPACK/1000*200 = 180	kWh					Electric energy 200 kWh/Mg fresh pasta
Transport, freight, lorry 7.5-16 metric ton, EURO5 {RER}| transport, freight, lorry 7.5-16 metric	HAWF*60 = 4.81E4	kgkm					TR HAWF - Bracciano (RM)- Roma: 60 km
Transport, freight, lorry 3.5-7.5 metric ton, EURO5 {RER}| transport, freight, lorry 3.5-7.5 metri	DETER*83 = 1.17	kgkm					TR Detergent - Nera Montoro (TR)-RM: 83 km
Transport, freight, lorry 3.5-7.5 metric ton, EURO5 {RER}| transport, freight, lorry 3.5-7.5 metri	OLUBR*103 = 2.74	kgkm					TR Lubrificating oil -Terni -RM: 103 km

Emissions to air	Sub-compartment	Amount	Unit	Distrit	SD2 or	Min	Max	Comment
Water	low. pop., long-term	85.7	kg	Undet				Water evaporated at the extruder
Water	low. pop., long-term	143.2	kg	Undet				Water evaporated to reduce the moisture of fresh pasta to 24% w/w (see Table 4).
Ethane, 1,1,1,2-tetrafluoro-, HFC-134a	low. pop., long-term	PFPACK*FGY*.04 = 23.4	mg					Fugitive gas leakage (R404)
Ethane, 1,1,1-trifluoro-, HFC-143a	low. pop., long-term	PFPACK*FGY*.52 = 305	mg					
Ethane, pentafluoro-, HFC-125	low. pop., long-term	PFPACK*FGY*.44 = 258	mg					

Outputs to technosphere: Waste and emissions to treatment	Amount	Unit	Distribu	SD2 o	Min	Max	Comment
Wastewater, from residence {RoW}| treatment of, capacity 1.1E10l/year | Cut-off, S	PFPACK/1000*72 = 64.9	l					72 L/Mg of fresh pasta
Biowaste {RoW}| treatment of biowaste by anaerobic digestion | Cut-off, S	ORGW*.255 = 4.62	kg					Organic Waste disposal (Table 8)
Sludge from pulp and paper production {Europe without Switzerland}| treatment of sludge from pulp and paper prc	ORGW*.31 = 5.62	kg					"
Raw sewage sludge {RoW}| treatment of, municipal incineration | Cut-off, S	ORGW*.18 = 3.26	kg					"S
Biowaste {RoW}| treatment of biowaste, industrial composting | Cut-off, S	ORGW*.255 = 4.62	kg					"

Table S10. Percentage fraction of the processing and packaging materials discarded, as collected in the fresh pasta factory examined in this work.

Processing and Packaging Materials discarded	Discarded Fraction (%)
Paper bags for wheat flour	1.0
Dough waste	0.4
Fresh pasta waste	1.5
Primary packaging	
PE bags	3.0
PE bag label	0.5
Secondary packaging	
Cartons (CAW)	1.0
Carton label	0.3
Scotch tape	0.3
Tertiary packaging	
Damaged wooden pallet	0.2
Wooden pallet label waste	0.2
Wooden pallet stretch & shrink film waste	0.2

Table S11. Total material balance of the packaging process of the HABW fresh pasta. All symbols were listed in the Nomenclature section.

**Step**	**Primary packaging**									
**Stream**	**PP**	**SPP**	**N_{2L}**	**CO_{2L}**		**PE**	**SPE**	**ES**	**SES**	**PFS**
	(kg)	(kg)	(kg)	(kg)	no.	(kg)	(kg)	(kg)	(kg)	(kg)
**Total**	915.0	13.7	9.0	4.5	1803	29.2	0.9	1.8	0.01	945.0
**Step**	**Secondary packaging**									
**Stream**		**CA**	**SCA**	**EC**	**SEC**	**SC**	**SCW**	**PFC**		
	no.	(kg)	(kg)	(kg)	(kg)	(kg)	(kg)	(kg)		
**Total**	150	85.0	0.8	0.3	0.001	1.1	0.003	1030.4		
**Step**	**Tertiary packaging**									
**Stream**		**PAL**	**SPAL**		**EP**	**SEP**	**FP**	**SFP**	**PFP**	
	n°	(kg)	(kg)	n°	(kg)	(kg)	(kg)	(kg)	(kg)	
**Total**	3.8	82.8	0.2	7.5	0.023	0.00005	2.574	0.005	1115.6	

Table S12. Inventory associated with the PE bag production step.

Products

Outputs to technosphere: Products and co-products	Amount	Unit	Quantity	Allocatio	Waste type	Category	Comment
FP-4 PE bag	1	kg	Mass	100 %	PE	Food\Cereal produ	This process allowed the manufacture of the primary packages for fresh pasta.
Add							

Outputs to technosphere: Avoided products	Amount	Unit	Distribution	SD2 or 2SD	Min	Max	Comment
Add							

Inputs

Inputs from nature	Sub-compartment	Amount	Unit	Distribution	SD2 or 2SD	Min	Max	Comment
Add								

Inputs from technosphere: materials/fuels	Amount	Unit	Distrit	SD2 (	Min	Max	Comment
Polyethylene, low density, granulate {RER}| production | Cut-off, S	1/0.976 = 1.02	kg					
Add							

Inputs from technosphere: electricity/heat	Amount	Unit	Distrib	SD2	Mi	Ma	Comment
Extrusion, plastic film {RER}| extrusion, plastic film | Cut-off, S	1/0.976 = 1.02	kg					
Transport, freight, lorry 7.5-16 metric ton, EURO5 {RER}| transport, freight, lorry 7.5-16 metric	1/0.976*150 = 154	kgkm					TR PE granules PS -Avezzano (AQ): 150 km

Outputs to technosphere: Waste and emissions to treatment	Amount	Unit	Distribut	SD2 or	Min	Max	Comment
Waste polyethylene {GLO}| treatment of waste polyethylene, open burning | Cut-off, S	1/.976-1 = 0.0246	kg					

Table S13. Inventory associated with the paper label production step.

Products

Outputs to technosphere: Products and co-products	Amount	Unit	Quantity	Allocatio	Waste type	Category	Comment
FP-5 paper label	1	kg	Mass	100 %	Paper	Paper+ B...\Market	Paper labels for I, II, & III packages
Add							

Outputs to technosphere: Avoided products	Amount	Unit	Distribution	SD2 or 2SD	Min	Max	Comment
Add							

Inputs

Inputs from nature	Sub-compartment	Amount	Unit	Distribution	SD2 or 2SD	Min	Max	Comment
Add								

Inputs from technosphere: materials/fuels	Amount	Unit	Distrit	SD2 (	Min	Max	Comment
Graphic paper, 100% recycled {RER}| production | Cut-off, S	1	kg	Unde				
Add							

Inputs from technosphere: electricity/heat	Amount	Unit	Distrib	SD2	Mi	Ma	Comment
Printed paper {Europe without Switzerland}| operation, printer, laser, colour, per kg | Cut-off	1	kg	Undefi				
Transport, freight, lorry 7.5-16 metric ton, EURO5 {RER}| transport, freight, lorry 7.5-16 metric	1*250 = 250	kgkm					TR Paper for labels PS-FG: 250 km

Table S14. Inventory associated with the carton production step.

Products							
Outputs to technosphere: Products and co-products	Amount	Unit	Quantity	Allocatio	Waste type	Category	Comment
FP-6 carton	1	kg	Mass	100 %	Cardboard	P...\Transformation	Production of cartons
Add							

Outputs to technosphere: Avoided products	Amount	Unit	Distribution	SD2 or 2SD	Min	Max	Comment
Add							

Inputs								
Inputs from nature	Sub-compartment	Amount	Unit	Distribution	SD2 or 2SD	Min	Max	Comment
Add								

Inputs from technosphere: materials/fuels	Amount	Unit	Distrit	SD2 (	Min	Max	Comment
Corrugated board box {RER}| production | Cut-off, S	1	kg	Unde				
Add							

Inputs from technosphere: electricity/heat	Amount	Unit	Distrib	SD2	Mi	Ma	Comment
Folding boxboard carton {RER}| market for folding boxboard carton | Cut-off, S	1	kg	Undefi				
Transport, freight, lorry 7.5-16 metric ton, EURO5 {RER}| transport, freight, lorry 7.5-16 metric	1*250 = 250	kgkm					TS: Raw Material x Cartons PF-FG: 250 km

Table S15. Inventory associated with the scotch tape production step.

Products

Outputs to technosphere: Products and co-products	Amount	Unit	Quantity	Allocatio	Waste type	Category	Comment
FP-7 scotch tape	1	kg	Mass	100 %	PP	Pl...\Transformation	Scotch tape x cartons
Add							

Outputs to technosphere: Avoided products	Amount	Unit	Distribution	SD2 or 2SD	Min	Max	Comment
Add							

Inputs

Inputs from nature	Sub-compartment	Amount	Unit	Distribution	SD2 or 2SD	Min	Max	Comment
Add								

Inputs from technosphere: materials/fuels	Amount	Unit	Distrit	SD2 (	Min	Max	Comment
Polypropylene, granulate {RER}| production | Cut-off, S	1/.976 = 1.02	kg					
Add							

Inputs from technosphere: electricity/heat	Amount	Unit	Distrib	SD2	Mi	Ma	Comment
Extrusion, plastic film {RER}| extrusion, plastic film | Cut-off, S	1/.976 = 1.02	kg					
Transport, freight, lorry 7.5-16 metric ton, EURO5 {RER}| transport, freight, lorry 7.5-16 metric	1/0.976*150 = 154	kgkm					TS: PE granules PS-FG: 150 km
Acrylic binder, without water, in 34% solution state {RER}| acrylic binder production, product	1/30 = 0.0333	kg					tape thichness 300 micron, acrylic one 10 micron

Outputs to technosphere: Waste and emissions to treatment	Amount	Unit	Distribut	SD2 or 2	Min	Max	Comment
Waste polypropylene {GLO}| treatment of waste polypropylene, open burning | Cut-off, S	1/0.976-1 = 0.0246	kg					

Table S16. Inventory associated with the EPAL wooden pallet production step.

Products

Outputs to technosphere: Products and co-products	Amount	Unit	Quantity	Allocatio	Waste type	Category	Comment
FP-8 EPAL Pallet	22	kg	Mass	100 %	Wood	Pl...\Transformation	
Add							

Outputs to technosphere: Avoided products	Amount	Unit	Distribution	SD2 or 2SD	Min	Max	Comment
Add							

Inputs

Inputs from nature	Sub-compartment	Amount	Unit	Distribution	SD2 or 2SD	Min	Max	Comment
Add								

Inputs from technosphere: materials/fuels	Amount	Unit	Distrit	SD2 (	Min	Max	Comment
EUR-flat pallet {RER}| market for EUR-flat pallet | Cut-off, S	1	p	Unde				
Add							

Inputs from technosphere: electricity/heat	Amount	Unit	Dis	SD	Mi	Ma	Comment
Transport, freight, lorry 16-32 metric ton, EURO5 {RER}| transport, freight, lorry 16-32 metric	22*100 = 2.2E3	kgkm					TR Wood x Pallet PS-wood pallet managing center: 100 km

Table S17. Inventory associated with the shrink & shrank PE film production step.

Products							
Outputs to technosphere: Products and co-products	Amount	Unit	Quantity	Allocatio	Waste type	Category	Comment
FP- 9 PE film x pallet	1	kg	Mass	100 %	PE	Pl...\Transformation	
Add							

Outputs to technosphere: Avoided products	Amount	Unit	Distribution	SD2 or 2SD	Min	Max	Comment
Add							

Inputs								
Inputs from nature	Sub-compartment	Amount	Unit	Distribution	SD2 or 2SD	Min	Max	Comment
Add								

Inputs from technosphere: materials/fuels	Amount	Unit	Distrit	SD2 (	Min	Max	Comment
Polyethylene, low density, granulate {RER}| production | Cut-off, S	1/.976 = 1.02	kg					
Add							

Inputs from technosphere: electricity/heat	Amount	Unit	Dis	SE	Mi	Ma	Comment
Extrusion, plastic film {RER}| extrusion, plastic film | Cut-off, S	1/0.976 = 1.02	kg					
Transport, freight, lorry 7.5-16 metric ton, EURO5 {RoW}| transport, freight, lorry 7.5-16 metri	1/0.976*150 = 154	kgkm					TR PE granules PS-FG: 150 km

Outputs to technosphere: Waste and emissions to treatment	Amount	Unit	Distribu	SD2 or 2	Min	Max	Comment
Waste polyethylene {GLO}| treatment of waste polyethylene, open burning | Cut-off, S	1/.976-1 = 0.0246	kg					
Add							

Table S18. Assembly of the primary packaging for fresh pasta.

Name	Status	Comment
FP- 13 Assembly of Primary Packaging	Finished	Assemblaggio del Pack I in MAP

Materials/Assemblies	Amount	Unit	Distribution	SD2 or 2SD	Min	Max	Comment
FP-4 PE bag	15.7	g	Triangle		15.4	16	
FP-5 paper label	1	g	Undefined				
Add							

Processes	Amount	Unit	Distribu	SD2 or 2S	Min	Max	Comment
Transport, freight, lorry 7.5-16 metric ton, EURO5 {RER}| transport, freight, lorry 7.5-16 metric tc	15.7/1000*106 = 1.66	kgkm					TR PE bags- Avezzano (AQ) - Roma: 106 km
Transport, freight, lorry 7.5-16 metric ton, EURO5 {RER}| transport, freight, lorry 7.5-16 metric tc	1/1000*65 = 0.065	kgkm					TR Labels- Nettuno (RM) - Roma: 65 km

Table S19. Assembly of the secondary packaging for fresh pasta.

Name	Status	Comment
FP- 14 Assembly of Secondary Packaging	Finished	Assembly of Secondary Packages for fresh pasta

Materials/Assemblies	Amount	Unit	Distribution	SD2 or 2SD	Min	Max	Comment
FP-6 carton	560	g	Triangle		555	565	
FP-5 paper label	2	g	Undefined				
FP-7 scotch tape	7	g	Undefined				
Add							

Processes	Amount	Unit	Distribu	SD2 or 2S	Min	Max	Comment
Transport, freight, lorry 7.5-16 metric ton, EURO5 {RER}| transport, freight, lorry 7.5-16 metric tc	560/1000*65 = 36.4	kgkm					TR Cartons - Nettuno (RM)-Roma: 65 km
Transport, freight, lorry 7.5-16 metric ton, EURO5 {RER}| transport, freight, lorry 7.5-16 metric tc	7/1000*100 = 0.7	kgkm					TR Scotch tape: Avezzano (AQ)-Roma: 100 km
Transport, freight, lorry 7.5-16 metric ton, EURO5 {RER}| transport, freight, lorry 7.5-16 metric tc	2/1000*65 = 0.13	kgkm					TR Paper labels- Nettuno (RM) - Roma: 65 km

Table S20. Assembly of the tertiary packaging for fresh pasta.

Name	Status	Comment
FP- 15 Assembly of Tertiary Packaging	Finished	Assembly of III Packaging in EPAL wood pallet

Materials/Assemblies	Amount	Unit	Distribution	SD2 or 2SD	Min	Max	Comment
FP-8 EPAL Pallet	22	kg	Triangle		21	23	
Add							

Processes	Amount	Unit	Distribu	SD2 or 2S	Min	Max	Comment
Transport, freight, lorry 7.5-16 metric ton, EURO5 {RER}| transport, freight, lorry 7.5-16 metric tc	22*30 = 660	kgkm					TR Pallet: EPMC Pomezia (RM)-Roma: 30 km

Table S21. Assembly of primary and secondary packages for fresh pasta.

Name	Status	Comment
FP - 16 Assembly of I & II packages	Finished	Assembly of I & II packaging (12 PE bags da 500 g in a carton)

Materials/Assemblies	Amount	Unit	Distribution	SD2 or 2SD	Min	Max	Comment
FP- 13 Assembly of Primary Packaging	12	p	Undefined				
FP- 14 Assembly of Secondary Packaging	1	p	Undefined				

Table S22. Assembly of primary, secondary, and tertiary packages for fresh pasta.

Name	Status	Comment
FP - 17 Assembly of I, II & III packages	Finished	Assembly of I, II & III Pack.s (40 carton xpallet + PE film+Labels

Materials/Assemblies	Amount	Unit	Distribution	SD2 or 2SD	Min	Max	Comment
FP - 16 Assembly of I & II packages	40	p	Undefined				
FP- 9 PE film x pallet	684	g	Undefined				
FP-5 paper label	2*3.11 = 6.22	g					
Add							

Processes	Amount	Unit	Distribut	SD2 or 2S	Min	Max	Comment
Transport, freight, lorry 7.5-16 metric ton, EURO5 {RoW}| transport, freight, lorry 7.5-16 metric t	684/1000*100 = 68.4	kgkm					TR Film x pallet - Avezzano (AQ)-Roma: 100 km
Transport, freight, lorry 7.5-16 metric ton, EURO5 {RoW}| transport, freight, lorry 7.5-16 metric t	2*3.11/1000*65 = 0.404	kgkm					TR Labels x pallet -Nettuno (RM)-Roma: 65 km

Table S23. Assembly of fresh pasta (1 Functional Unit).

Name	Status	Comment
FP - 18 Assembly of Fresh Pasta	Finished	Assemby of 1 Functional Unit: 500-g fresh pasta in modified atmosphera

Materials/Assemblies	Amount	Unit	Distribution	SD2 or 2SD	Min	Max	Comment
FP- 3 Fresh Pasta from HAWF, at packaging	0.5	kg	Undefined				
Nitrogen, liquid {RER}| market for | Cut-off, S	5	g	Undefined				
Carbon dioxide, liquid {RER}| market for | Cut-off, S	2.5	g	Undefined				
Add							

Processes	Amount	Unit	Distribut	SD2 or 2S	Min	Max	Comment
Transport, freight, lorry 7.5-16 metric ton, EURO5 {RER}| transport, freight, lorry 7.5-16 metric tc	(5+2.5)/1000*95 = 0.713	kgkm					TR Liquid N2 + CO2-Nera Montoro (TR)-Roma: 95 km

Table S24. Inventory associated with the use phase.

Products

Outputs to technosphere: Products and co-products	Amount	Unit	Quantity	Allocatio	Category	Comment
FP- 19 Use Phase	RFP = 1	kg	Mass	100 %	Ot...\Consumer Use	Pasta fresca pastorizzata cruda
FP - Cooked Pasta waste	Pcotta*PW = 0.034	kg	Mass	0 %	Ot...\Consumer Use	
Add						

Outputs to technosphere: Avoided products	Amount	Unit	Distribution	SD2 or 2SD	Min	Max	Comment
Add							

Inputs

Inputs from nature	Sub-compartment	Amount	Unit	Distribution	SD2 or 2SD	Min	Max	Comment
Add								

Inputs from technosphere: materials/fuels	Amount	Unit	Distrit	SD2 (	Min	Max	Comment
FP- 3 Fresh Pasta from HAWF, at packaging	0	kg	Unde				
Tap water {Europe without Switzerland}| market for | Cut-off, S	RFP*WFPR = 10	kg					
Salt, white, for human consumption (sea, igneous or rock), no enrichment, processed in FR | Ambient (Ic	Salt*RFP = 100	g					
Add							

Inputs from technosphere: electricity/heat	Amount	Unit	Dis	SI	M	Ma	Comment
Electricity, low voltage {IT}| market for | Cut-off, S	EC*0.17 = 0.336	kWh					It referred to the energy consumed (17 % by electric hob) to cook 1 kg of dry pasta (UNAFPA, 2018).
Heat, central or small-scale, natural gas {Europe without Switzerland}| market for heat, central or small-scal	EC*0.83 = 1.64	kWh					It referred to the energy consumed (83 % by gas-fired hob) to boil 10 L of water and cook 1 kg of dry pasta for 10 min (UNAFPA, 2018).
Electricity, low voltage {IT}| market for | Cut-off, S	EFRIDGE = 2.46	kWh					Electricity consumed for preserving fresh pasta in fridge

Outputs

Emissions to air	Sub-compartment	Amount	Unit	Distril	SD2 or	Min	Max	Comment
Water	low. pop., long-term	WE = 0.85	kg					Acqua evaporata durante la cottura
Nitrogen, atmospheric	low. pop., long-term	10	g	Unde				N2 freed as 1-kg fresh pasta bags (2 packages) are opened
Carbon dioxide, fossil	low. pop., long-term	5	g	Unde				Idem

Outputs to technosphere: Waste and emissions to treatment	Amount	Unit	Dis	SD	Min	Ma	Comment
Biowaste {CH}| treatment of biowaste by anaerobic digestion | Conseq, S	Pcotta*PW*0.255 = 0.00867	kg					Pasta waste disposed as oeganic wasteS
Biowaste {CH}| treatment of biowaste, industrial composting | Cut-off, S	Pcotta*PW*0.255 = 0.00867	kg					see Table 8
Sludge from pulp and paper production {Europe without Switzerland}| treatment of sludge from pulp and paper pro	Pcotta*PW*.31 = 0.0105	kg					Idem
Raw sewage sludge {RoW}| treatment of, municipal incineration | Cut-off, S	Pcotta*PW*.18 = 0.00612	kg					Idem
Wastewater, from residence {RoW}| treatment of, capacity 1.1E10l/year | Cut-off, S	PWATER = 8.5	l					Pasta water disposal in the kitchen sink

Table S25. Waste scenario for plastic packaging wastes (PLW).

Products

Waste specification	Amount	Unit	Category	Comment
FP - 10 Plastic Waste Scenarios	1	kg	Others	

Inputs

Inputs from technosphere: materials/fuels	Amount	Unit	Distrit	SD2 ‹	Min	Max	Comment
Add							

Inputs from technosphere: electricity/heat	Amount	Unit	Dis	SI	M	Mi	Comment
Add							

Outputs

Materials and/or waste types separated from waste stream	Material / Waste type	Percentage	Comment
Add			

Waste streams remaining after separation	Percentage	Comment
Waste polyethylene {RoW}| treatment of waste polyethylene, municipal incineration | Cut-off, S	47 %	see Table 8
PE (waste treatment) {GLO}| recycling of PE | Cut-off, S	45.6 %	Idem
Waste polyethylene {RoW}| treatment of waste polyethylene, sanitary landfill | Cut-off, S	7.4 %	Idem

Table S26. Waste scenario for cardboard and paper packaging wastes (PCW).

Waste specification	Amount	Unit	Category	Comment
FP - 11 Paper&Cardboard Waste Scenarios	1	kg	Others	

Inputs

Inputs from technosphere: materials/fuels	Amount	Unit	Distrit	SD2 (	Min	Max	Comment
Add							

Inputs from technosphere: electricity/heat	Amount	Unit	Dis	SI	M	Ma	Comment
Add							

Outputs

Materials and/or waste types separated from waste stream	Material / Waste type	Percentage	Comment
Add			

Waste streams remaining after separation	Percentage	Comment
Waste paperboard {RoW}| treatment of, municipal incineration | Cut-off, S	7.6 %	see Table 8
Paper (waste treatment) {GLO}| recycling of paper | Cut-off, S	80.8 %	Idem
Waste paperboard {RoW}| treatment of, sanitary landfill | Cut-off, S	11.6 %	Idem

Table S27. Waste scenario of wooden pallet wastes (PALW**).**

Products				
Waste specification	Amount	Unit	Category	Comment
FP - 12 Pallet Waste Scenarios	1	kg	Others	

Inputs

Inputs from technosphere: materials/fuels	Amount	Unit	Distrit	SD2 (	Min	Max	Comment
Add							

Inputs from technosphere: electricity/heat	Amount	Unit	Dis	Sl	M	Mi	Comment
Add							

Outputs

Materials and/or waste types separated from waste stream	Material / Waste type	Percentage	Comment
Add			

Waste streams remaining after separation	Percentage	Comment
Waste wood, untreated {RoW}| treatment of waste wood, untreated, municipal incineration | Cut-off, S	2.1 %	see Table 8
Waste wood, untreated {IT}| market for waste wood, untreated | Cut-off, S	63.1 %	Idem
Waste wood, untreated {RoW}| treatment of, sanitary landfill | Cut-off, S	34.8 %	Idem

Table S28. End-of-life of primary packaging material wastes (EoL-1pack).

Name	Status	Comment
FP - 20 EoL Primary Packaging	Finished	

Referring to assembly	Amount	Unit	Comment
FP- 13 Assembly of Primary Packaging	1	p	

Processes	Amount	Unit	Distribut	SD2 or 2S	Min	Max	Comment
Transport, freight, lorry 7.5-16 metric ton, EURO5 {RoW}| transport, freight, lorry 7.5-16 metric t	16.7/1000*50 = 0.835	kgkm					TR Plastic and Paper wastes from FPFG, CH to - Waste Collection Centre: 50 km

Add

Waste scenarios	Percentage	Comment
FP - 10 Plastic Waste Scenarios	94.03 %	
FP- Paperboard Waste Disposal Scenario	5.97 %	

Table S29. End-of-life of secondary packaging material wastes (EoL-2pack).

Name	Status	Comment
FP - 21 EoL Secondary Packaging	None	

Referring to assembly	Amount	Unit	Comment
FP- 14 Assembly of Secondary Packaging	1	p	

Processes	Amount	Unit	Distribut	SD2 or 2S	Min	Max	Comment
Transport, freight, lorry 7.5-16 metric ton, EURO5 {RoW}| transport, freight, lorry 7.5-16 metric t	569/1000*50 = 28.4	kgkm					TR Paper&Cardboard and plastic waste from DC to waste collection center WCC: 50 km

Add

Waste scenarios	Percentage	Comment
FP - 10 Plastic Waste Scenarios	1.23 %	
FP - 11 Paper&Cardboard Waste Scenarios	98.77 %	

Table S30. End-of-life of tertiary packaging material wastes (EoL-3pack**).**

Name	Status	Comment
FP - 22 EoL Tertiary Packaging	Finished	

Referring to assembly	Amount	Unit	Comment
FP- 15 Assembly of Tertiary Packaging	1	p	

Processes	Amount	Unit	Distribu	SD2 or 2S	Min	Max	Comment
Transport, freight, lorry 7.5-16 metric ton, EURO5 {RoW}| transport, freight, lorry 7.5-16 metric t	22*.998*100 = 2.2E3	kgkm					TR Reusable Pallets from DCs to EPAL Pallet managing Center (EPCM) Pomezia (RM): 100 km
Transport, freight, lorry 7.5-16 metric ton, EURO5 {RoW}| transport, freight, lorry 7.5-16 metric t	22*.002*50 = 2.2	tkm					TR Broken Palles from DCs to EPCM: 50 km
Add							

Waste scenarios	Percentage	Comment
FP - 12 Pallet Waste Scenarios	0.2 %	D
Add		

Disassemblies	Percentage	Comment
Add		

Reuses	Percentage	Comment
FP - 24 Pallet reuse	99.8 %	

Table S31. End-of-life scenario for the primary, secondary and tertiary packaging material wastes, excluding wooden pallets (EoL-1-3pack**).**

Name	Status	Comment
FP - 23 EoL I, II & III Packaging	Finished	Not including wood pallets

Referring to assembly	Amount	Unit	Comment
FP - 17 Assembly of I, II & III packages	1	p	

Processes	Amount	Unit	Distr	SD2 or 2S	Min	Max	Comment
Transport, freight, lorry 7.5-16 metric ton, EURO5 {RoW}| transport, freight, lorry 7.5-16 metric t	31.485*50 = 1.57E3	kgkm					TR Paper and plastic wastes from DCs to WCC: 50 km
Add							

Waste scenarios	Percentage	Comment
FP - 10 Plastic Waste Scenarios	27.06 %	
FP - 11 Paper&Cardboard Waste Scenarios	72.94 %	

Table S32. Reuse phase of the wooden pallet (Pallet reuse).

Name			Status	Comment
FP - 24 Pallet reuse			Finished	
Referring to assembly	Amount	Unit		Comment
FP- 15 Assembly of Tertiary Packaging	1	p		

Table S33. Life cycle of HABWF fresh pasta (LC-FP).

Name			Status				Comment
FP-Life Cycle of Fresh Pasta			Finished				
**Assembly**	**Amount**	**Unit**	**Distributioı**	**SD2 or 2SD**	**Min**	**Max**	**Comment**
FP - 18 Assembly of Fresh Pasta	2	p	Undefined				Two 500-g bags

Processes	Amount	Unit	Distr	SD2 or 2S	Min	Max	Comment
Transport, freight, lorry with refrigeration machine, 7.5-16 ton, EURO5, R134a refrigerant, freezi	293.48/240*.5*2*100 = 122	kgkm					TR Palletized fresh pasta from Rome to DCs: 100 km
FP- 19 Use Phase	1	kg	Und				
Add							

Waste/Disposal scenario	Comment

Additional life cycles	Number	Distributioı	SD2 or 2SD	Min	Max	Comment
FP - Life Cycle of I, II & III packaging	1/240 = 0.00417					

Table S34. Life cycle of the primary, secondary, and tertiary packaging materials (LC-1-3pack).

Name	Status	Comment
FP - Life Cycle of I, II & III packaging	Finished	

Assembly	Amount	Unit	Distributioı	SD2 or 2SD	Min	Max	Comment
FP - 17 Assembly of I, II & III packages	1	p	Undefined				

Processes	Amount	Unit	Distr	SD2 or 2!	Min	Max	Comment
Add							

Waste/Disposal scenario	Comment
FP - 23 EoL I, II & III Packaging	

Additional life cycles	Number	Distributioı	SD2 or 2SD	Min	Max	Comment
FP-Life Cycle of Wooden Pallet	1	Undefined				

Table S35. Life cycle of the wooden pallet (LC-3pack).

Name	Status	Comment
FP-Life Cycle of Wooden Pallet	None	

Assembly	Amount	Unit	Distributioı	SD2 or 2SD	Min	Max	Comment
FP- 15 Assembly of Tertiary Packaging	1	p	Undefined				

Processes	Amount	Unit	Distributioı	SD2 or 2SD	Min	Max
Add						

Waste/Disposal scenario	Comment
FP - 22 EoL Tertiary Packaging	

Table S36. Inventory associated with the cultivation phase of a typical bread wheat in Central Italy.

Products

Outputs to technosphere: Products and co-products	Amount	Unit	Quantity	Allocation %	Waste type	Category	Comment
FP GT -1 Soft Wheat Grain	CropYield = 7.26E3	kg	Mass	92.5 %	Biopolymer	A...\Transformation	
STRAW SW	STRAW/SSSTRAW = 12.4	ton	Mass	7.5 %	Biopolymer	A...\Transformation	

Add

Outputs to technosphere: Avoided products	Amount	Unit	Distribution	SD2 or 2SD	Min	Max	Comment

Add

Inputs

Inputs from nature	Sub-compartment	Amount	Unit	Distribution	SD2 or 2SD	Min	Max	Comment
Occupation, annual crop, IT		AREA = 1	ha a					

Add

Inputs from technosphere: materials/fuels	Amount	Unit	Distrit	SD2 (	Min	Max	Comment
Urea {RER}| urea production | Cut-off, S	UREA*AREA = 150	kg					See Table 1
Diammonium phosphate {RER}| diammonium phosphate production | Cut-off, S	FOSFBIAM*AREA = 200	kg					"
Ammonium nitrate {RER}| ammonium nitrate production | Cut-off, S	NITRAMM*AREA = 150	kg					"
Pesticide, unspecified {RER}| production | Cut-off, U	1.8*AREA = 1.8	kg					Fungicide (1 L/ha) + Herbicide (0.8 L/ha)
Diesel {Europe without Switzerland}| market for | Cut-off, S	70*AREA = 70	kg					See Table 1
Lubricating oil {RER}| market for lubricating oil | Cut-off, S	0.2*AREA = 0.2	kg					"
Wheat seed, for sowing {GLO}| production | Cut-off, S	Seed*AREA = 250	kg					"

Inputs from technosphere: electricity/heat	Amount	Unit	Dis	Sl	M	Ma	Comment
Transport, freight, lorry 3.5-7.5 metric ton, EURO5 {RoW}| transport, freight, lorry 3.5-7.5 metric ton, EURO5 |	Seed*AREA*55 = 1.38E4	kgkm					Transport seedsi VT-Anguillara Sabazia: 55 km
Transport, freight, lorry 7.5-16 metric ton, EURO5 {RER}| transport, freight, lorry 7.5-16 metric ton, EURO5 | C	(UREA+FOSFBIAM+NITRAMM)*AREA	kgkm					TR fertilizers Varie-Anguillara Sabazia: 100 km
Transport, freight, light commercial vehicle {Europe without Switzerland}| market for transport, freight, light	(1.8+70+0.2)*AREA*25 = 1.8E3	kgkm					TR pest+diesel fuel+Lubr oil Varie-Angu. Sab.: 25 km
Transport, freight, lorry 7.5-16 metric ton, EURO5 {RER}| transport, freight, lorry 7.5-16 metric ton, EURO5 | C	STRAW/SSSTRAW*50 = 619	tkm					TR Paglia Ang Sab-Varie: 50 km

Add

Outputs

Emissions to air	Sub-compartment	Amount	Unit	Distril	SD2 or	Min	Max	Comment
Dinitrogen monoxide	low. pop., long-term	FN2OTOT = 3.82	kg					
Nitrogen monoxide, IT	low. pop., long-term	NOA = 4.59	kg					
Carbon dioxide, fossil	low. pop., long-term	CO2U = 55	kg					

Add

Emissions to water	Sub-compartment	Amount	Unit	Distributio	SD2 or 2SI	Min	Max	Comment
Nitrate, IT	groundwater, long	NITRL = 43.1	kg					
Phosphorus, IT	groundwater, long	PLEACH = 2.01	kg					

Table S37. Inventory associated with the milling phase of common wheat grains cultivated in Central Italy.

Products

Outputs to technosphere: Products and co-products	Amount	Unit	Quantity	Allocation %	Waste type	Category	Comment
FP GT - 2 Bread Wheat Flour, at industrial mill	HAWG*YHAWF = 5.3E3	kg	Mass	84 %	Biopolymer	Food\Cereal produ	Economic allocation factors (UNAFTA, 2
FP GT -Pelletized Middlings	HAWG*YWFP = 2.21E3	kg	Mass	16 %	Biopolymer	Food\Cereal produ	
FP GT -ISS	HAWG*YISS = 3.63	kg	Mass	0 %	Others	Food\Cereal produ	Impurities
Add							

Outputs to technosphere: Avoided products	Amount	Unit	Distribution	SD2 or 2SD	Min	Max	Comment
Add							

Inputs

Inputs from nature	Sub-compartment	Amount	Unit	Distribution	SD2 or 2SD	Min	Max	Comment
Add								

Inputs from technosphere: materials/fuels	Amount	Unit	Distrit	SD2 (	Min	Max	Comment
FP GT -1 Soft Wheat Grain	HAWG = 7.26E3	kg					MP
Tap water {Europe without Switzerland}| tap water production, underground water without treatment | (	HAWG*YMO = 238	kg					
FP-1 bis paper bags x flour	NSF*MSF*(1+SPSAF) = 24.6	kg					
Chlorine, liquid {RER}| chlorine production, liquid | Cut-off, S	DETER = 0.0123	kg					
Lubricating oil {RER}| market for lubricating oil | Cut-off, S	OLUBR = 0.0152	kg					
Add							

Inputs from technosphere: electricity/heat	Amount	Unit	Dis	SI	M	Ma	Comment
Electricity, medium voltage {IT}| electricity voltage transformation from high to medium voltage | Cut-off, S	147*HAWG/1000 = 1.07E3	kWh					147 kWh/Mg
Transport, freight, lorry 7.5-16 metric ton, EURO5 {RER}| transport, freight, lorry 7.5-16 metric ton, EURO5 | Cı	HAWG*20 = 1.45E5	kgkm					TR HAWF grains Anguillara Sabazia-Bracciano: 20 km
Transport, freight, lorry 16-32 metric ton, EURO5 {RER}| transport, freight, lorry 16-32 metric ton, EURO5 | Cu	NSF*MSF*(1+SSAF)*527 = 1.6E4	kgkm					TR paper bags Rho (MI)-Spoleto (PG): 527 km
Transport, freight, lorry 3.5-7.5 metric ton, EURO5 {RER}| transport, freight, lorry 3.5-7.5 metric ton, EURO5 | (	(HAWG*YISS+SSAF)*50 = 194	kgkm					TR ISS+paper bag waste: 50 km
Transport, freight, lorry 3.5-7.5 metric ton, EURO5 {RER}| transport, freight, lorry 3.5-7.5 metric ton, EURO5 | (	DETER/1000*112 = 0.00138	kgkm					TR Detergent- Nera Montoro (TR) -Bracciano: 112 kn
Transport, freight, lorry 3.5-7.5 metric ton, EURO5 {RER}| transport, freight, lorry 3.5-7.5 metric ton, EURO5 | (	OLUBR/1000*133 = 0.00203	kgkm					TR Lubrificating oil -Terni-Bracciano: 133 km
Transport, freight, lorry 7.5-16 metric ton, EURO5 {RER}| transport, freight, lorry 7.5-16 metric ton, EURO5 | Cı	HAWG*YWFP/1000*50 = 111	tkm					TR Milling by-products: MFG-CF: 50 km
Add							

Outputs

Emissions to air	Sub-compartment	Amount	Unit	Distril	SD2 or	Min	Max	Comment
Water	low. pop.	44.6	kg	Unde				see Table 3

Outputs to technosphere: Waste and emissions to treatment	Amount	Unit	Dis	SD:	Min	Ma	Comment
Inert waste, for final disposal {RoW}| treatment of inert waste, inert material landfill | Cut-off, S	HAWG*YISS = 3.63	kg					
Paper (waste treatment) {GLO}| recycling of paper | Cut-off, S	SSAF*.808 = 0.197	kg					
Waste paperboard {RoW}| treatment of, sanitary landfill | Cut-off, S	SSAF*.116 = 0.0283	kg					
Waste paperboard {Europe without Switzerland}| treatment of waste paperboard, municipal incineration | Cut-off, S	SSAF*.076 = 0.0185	kg					

Table S38. Inventory associated with the fresh pasta production step using type 00 bread wheat flour.

Products

Outputs to technosphere: Products and co-products	Amount	Unit	Quantity	Allocation %	Waste type	Category	Comment
FPGT- 3 Fresh Pasta from BWF, at packaging	PFPACK = 5.98E3	kg	Mass	100 %	Compost	Food\Cereal produ	
FPGT-SI-SPP	ORGW = 117	kg	Mass	0 %	Biopolymer	Food\Cereal produ	scarti impasto+pasta fresca pastorizzata
Add							

Outputs to technosphere: Avoided products	Amount	Unit	Distribution	SD2 or 2SD	Min	Max	Comment
Add							

Inputs

Inputs from nature	Sub-compartment	Amount	Unit	Distribution	SD2 or 2SD	Min	Max	Comment
Add								

Inputs from technosphere: materials/fuels	Amount	Unit	Distrit	SD2 (	Min	Max	Comment
FP GT - 2 Bread Wheat Flour, at industrial mill	HAWF = 5.3E3	kg					
Tap water {Europe without Switzerland}| market for | Cut-off, S	HAWF*WFR = 1.32E3	kg					Acqua per Impasto
Water, deionised {Europe without Switzerland}| water production, deionised | Cut-off, S	HAWF*YVPAST = 334	kg					Per produrre vapore per pastorizzare la pasta fresca
Chlorine, liquid {GLO}| production | Cut-off, S	DETER = 0.0933	kg					
Lubricating oil {RER}| market for lubricating oil | Cut-off, S	OLUBR = 0.176	kg					
Add							

Inputs from technosphere: electricity/heat	Amount	Unit	Dis	SI	M	Ma	Comment
Heat, central or small-scale, natural gas {Europe without Switzerland}| heat production, natural gas, at boile	HAWF*YPP/1000*78.8 = 479	kWh					
Electricity, medium voltage {IT}| electricity voltage transformation from high to medium voltage | Cut-off, S	HAWF*YPP/1000*147.8 = 898	kWh					
Transport, freight, lorry 7.5-16 metric ton, EURO5 {RER}| transport, freight, lorry 7.5-16 metric ton, EURO5 | Cu	HAWF*150 = 7.95E5	kgkm					TR HAWF - Spoleto- Roma: 150 km
Transport, freight, lorry 3.5-7.5 metric ton, EURO5 {RER}| transport, freight, lorry 3.5-7.5 metric ton, EURO5 | C	DETER*83 = 7.75	kgkm					TR Detergente - Nera Montoro (TR)-RM: 83 km
Transport, freight, lorry 3.5-7.5 metric ton, EURO5 {RER}| transport, freight, lorry 3.5-7.5 metric ton, EURO5 | C	OLUBR*103 = 18.2	kgkm					TR Olio lubrificante -Terni -RM: 103 km

Outputs

Emissions to air	Sub-compartment	Amount	Unit	Distril	SD2 or	Min	Max	Comment
Water	low. pop., long-term	HAWF*YVTRAF = 98.6	kg					Water evaporated alle trafile
Water	low. pop., long-term	HAWF*YVASC = 753	kg					Water evaporated per ridurre al 24% p/p l'umidità della pasta Fresca
Ethane, pentafluoro-, HFC-125	low. pop., long-term	PFPACK*YFG*.44 = 1.71E3	mg					
Ethane, 1,1,1,2-tetrafluoro-, HFC-134a	low. pop., long-term	PFPACK*YFG*.04 = 156	mg					
Ethane, 1,1,1-trifluoro-, HFC-143a	low. pop., long-term	PFPACK*YFG*.52 = 2.02E3	mg					

Outputs to technosphere: Waste and emissions to treatment	Amount	Unit	Dis	SD.	Min	Ma	Comment
Wastewater, from residence {RoW}| treatment of, capacity 1.1E10l/year | Cut-off, S	PFPACK/1000*72 = 431	l					72 L/Mg of fresh pasta
Biowaste {RoW}| treatment of biowaste by anaerobic digestion | Cut-off, S	ORGW*.255 = 30	kg					
Sludge from pulp and paper production {Europe without Switzerland}| treatment of sludge from pulp and paper pro	ORGW*.31 = 36.4	kg					
Raw sewage sludge {RoW}| treatment of, municipal incineration | Cut-off, S	ORGW*.18 = 21.1	kg					
Biowaste {RoW}| treatment of biowaste, industrial composting | Cut-off, S	ORGW*.255 = 30	kg					
